# Visual, auditory, and audiovisual time-to-collision estimation among participants with age-related macular degeneration compared to a normal-vision group: The TTC-AMD study

**DOI:** 10.1371/journal.pone.0337549

**Published:** 2025-12-04

**Authors:** Patricia R. DeLucia, Daniel Oberfeld, Joseph K. Kearney, Melissa Cloutier, Anna M. Jilla, Avery Zhou, Stephanie Trejo Corona, Jessica Cormier, Audrey Taylor, Charles C. Wykoff, Robin Baurès

**Affiliations:** 1 Department of Psychological Sciences, Rice University, Houston, Texas, United States of America; 2 Section Experimental Psychology, Psychologisches Institut, Johannes Gutenberg-Universität Mainz, Mainz, Germany; 3 Department of Computer Science, The University of Iowa, Iowa city, Iowa, United States of America; 4 Department of Speech and Hearing Sciences, Lamar University, Beaumont, Texas, United States of America; 5 Retina Consultants of Texas, Retina Consultants of America, Houston, Texas, United States of America; 6 Davies Institute for Speech & Hearing, Katy, Texas, United States of America; 7 Univ. Toulouse, CNRS, CerCo, Toulouse, France; University of Wisconsin-Milwaukee, UNITED STATES OF AMERICA

## Abstract

Little is known about whether and to what degree people with different amounts of visual impairment rely on hearing instead of vision for mobility, particularly in judgments of collision. We measured how much importance was assigned to visual and auditory cues during time-to-collision judgments made by people with age-related macular degeneration (Impaired Vision Group; IV) compared to a control group without age-related macular degeneration (Normal Vision Group; NV). A virtual reality system simulated a roadway with an approaching vehicle viewed from the perspective of a pedestrian. Participants pressed a button to indicate the time the vehicle would reach them. The vehicle was presented visually only, aurally only, or both simultaneously. Standardized regression coefficients and general dominance weights indicated that time-to-collision (TTC) judgments were determined by both auditory and visual cues in both groups. In the vision-only modality condition, the relative importance of distance and optical size compared to TTC was higher in the IV group compared to the NV group, but with a relatively small effect size. In all modality conditions, the mean absolute error of TTC estimates was comparable between groups, and a multimodal advantage was not observed. Intraindividual variability was greater in the IV group only in the AV condition. The implication is that similar performance can be achieved through the use of different sources of information. Importantly, people with and without IV achieved similar performance but showed differences in the relative importance of different sensory sources of information. A comparison of two IV subgroups differing in severity suggested that simply having IV in both eyes is not sufficient to predict TTC estimation differences between people with IV and people without IV who have normal vision. Rather it appears to be the degree of bilateral visual impairment of the IV that matters.

## Introduction

More than 7 million individuals in the United States, and more than 250 million individuals world-wide, are blind or have low vision [1]. This incidence is increasing because both the absolute number, and proportion of older people globally, is increasing [2,3]. People with visual impairments experience decrements in social functioning and exhibit twice the rate of social isolation and depression compared to those comparable in age with normal vision [4]. More than half are not in the labor force [5]. To improve the independence and quality of life for people with vision loss, it is essential to consider ways to improve their mobility and thus their ability to avoid collisions, which is critical for daily activities such as navigation, driving a vehicle, and engaging in safe pedestrian behavior [6]. For example, crossing a street and moving indoors amidst obstacles is a meaningful challenge for the visually impaired [7–10]. Further, people with age-related macular degeneration (AMD) are at higher risk for falls, are less likely to travel or drive, are slower to detect traffic gaps while crossing streets; and they experience impairments in face and scene perception, reading, and many other daily activities [11]. Yet, there are few studies of collision perception with vision loss and, therefore, there remains a tremendous unmet need for additional prospective data analyzing tasks that are essential for mobility.

### Loss of central vision degrades collision judgments

Loss of central vision, such as is observed in severe cases of advanced AMD, is likely to impair the detection of information necessary for effective collision judgments and negatively affects mobility, including driving [11]. To recognize that an object is approaching on the basis of sight, and respond appropriately to avoid collision, observers must detect optical expansion [12]. This refers to the increase in an object’s optical (retinal) image size as it gets closer to the observer. Within this “looming” pattern is a sensory variable, *visual tau*, that can reliably provide accurate information about how much time remains until a collision would occur—time-to-collision (TTC), when certain conditions are met [13,14]. There is analogous TTC information in the auditory domain based on changes in sound intensity (*auditory tau*) [15]. For a direct approach (for tau variables for other trajectories, see [16]), visual expansion tau is derived from the ratio of the approaching object’s optical size to its instantaneous rate of optical expansion. People can use expansion tau to estimate TTC [17], but expansion tau is effective only if expansion rate is above detection threshold [12]. Thresholds for detecting looming of vehicles in simulated street-crossing scenarios were better when the approaching vehicles fell in foveal vision compared to even slight extrafoveal vision [12]. Loss of central vision and greater reliance on peripheral vision (e.g., preferred retinal locus; [18]), key features of AMD, result in higher looming thresholds and later detection of looming compared to intact central vision. Consequently, vehicle approach motion is detected later or less precisely, allowing less time to avoid a collision, for example, while crossing the street. Indeed, those with AMD had longer street-crossing judgment times and shorter safety margins than those with normal vision [9]. Compared to individuals with normal vision, those with AMD performed more poorly on driving tasks, shown by brake reaction time [19], gap selection, and lane keeping, and were rated as less safe drivers [20].

Although prior research on rehabilitation for visual impairments included mobility, many studies relied on qualitative or subjective measures (e.g., surveys), or did not focus on skills necessary for collision avoidance [10,21]. To be effective, rehabilitation and interventions must enhance the ability to differentiate whether a collision with another person will occur, as is essential in many ordinary tasks such as walking in busy shopping malls or train stations [22]. Strategies to help people with visual impairments avoid collisions include global navigation technologies, but these have not been effective at facilitating collision avoidance in the blind [23].

An alternative is sensory substitution in which sound is used in place of or to augment vision. However, most studies of auditory sensory substitution focused on auditory localization [24]. Studies of individuals with normal vision have reported that auditory information can be useful in collision perception [25,26], but research on collision judgments by people with visual impairment is sparse. Based on a study of six blind (no vision or light vision only) and 60 normally sighted individuals, [17] tentatively suggested that the TTC judgments of (filmed) approaching vehicles were comparable in accuracy between blind individuals using auditory information and normally sighted individuals using visual information. Accuracy was greater for blind than normally sighted when both used auditory information. This study did not measure collision judgments in individuals who have functional residual vision and consequently may combine information from both vision and audition. In another study [27], participants who had normal vision or who were visually impaired or blind, were presented with different vehicular gaps in real traffic on a real street and used a rating scale to report whether there was sufficient time to cross the street. Judgments of individuals with normal and impaired vision were comparable when they only saw, or both saw and heard sounds from the real traffic. When only hearing the traffic, those with blindness were less accurate than those with normal or impaired vision and all groups overestimated the gap time. The purpose of the current study was to measure whether and how visual and auditory information are used and integrated in collision judgments by individuals with central vision loss due to AMD, and determine the relative weights of different visual and auditory cues.

### Sensory substitution may not be used when there is residual vision

Sensory substitution, such as relying on audition instead of vision [6], has been studied extensively as a strategy to help those with visual impairment (e.g., [21]). However, prior studies typically focused on individuals with complete or nearly complete vision loss (e.g., [21]) rather than individuals with residual vision who account for more of the visually impaired population. This is relevant because deterioration in vision due to progressive, neurogenerative retinal disease that can lead to central vision loss, such as AMD, typically occurs gradually over time and rarely results in complete vision loss (e.g., [28]). This makes it important to study effects of vision loss when residual vision is available. Moreover, the leading cause of vision loss among older people is AMD, which typically leads to partial rather than complete vision loss (e.g., [27]).

It has been assumed that sensory substitution occurs when individuals lose visual function, that is, they compensate by relying more heavily on other intact modalities such as audition. For example, it has been stated that “*Generally, hearing becomes more important for navigation as vision decreases*” [29]. Studies have reported that blind individuals can outperform sighted individuals who use normal vision or wear a blindfold when tested on alternate modalities such as audition and touch [8,17,29,30], although actual data regarding such cross-modal enhancement are mixed [31]. For example, in people with early blindness who lack light perception, parts of the brain that process auditory motion can recruit brain areas that process visual motion [32]. However, prior studies focused on individuals with complete or nearly complete vision loss (e.g., congenitally blind; blind-folded sighted individuals). For example, the ability to detect a traffic gap during street-crossing typically was compared between full sight and total blindness [9]. In 2017, 7 million people had vision acuity loss (best corrected logMAR of 0.3 or greater), and 1 million of those had blindness (1.0 logMAR or greater; [1]). Whereas individuals with severe or complete vision loss have no alternative but to rely on other modalities, those with residual vision can continue to rely on vision. Cross-modal enhancement increases with greater loss, and auditory compensation may not emerge, or may emerge to varying degrees, when residual vision is present [33]. The implication is that those with residual vision may not benefit from auditory (or tactile) information to compensate for partial vision loss, and any benefits may depend on the severity of the loss. Further, people with equal visual impairment may vary in their ability to use different sensory information. Nevertheless, we note that a prior study of street-crossing showed that individuals who were blind (i.e., light perception) exhibited less effective street-crossing decisions when only auditory information was presented compared to individuals with normal or impaired vision [27]. The current study examined whether people with residual vision rely more strongly on audition instead of vision and how much each sensory modality contributes to collision judgments.

### Multisensory information integration includes reliable and heuristic cues

People use both reliable (consistently accurate) and heuristic (less reliably accurate) cues when making collision judgments. For example, in our prior study of audiovisual TTC judgments in normally sighted participants [34], we measured weights assigned by participants to auditory and visual cues that reliably provided accurate TTC information, specifically the presented auditory and visual TTCs [13–15]. We also measured weights assigned to simpler heuristic cues, which are less reliably accurate and can be misleading in certain conditions. These included optical size (i.e., the pictorial depth cue of relative size) and sound pressure level (SPL) at the moment when the TTC judgment is made, which can influence TTC estimates [35–38]. In the “size-arrival effect” [35] observers reported that a large far approaching object with a relatively larger optical size appeared to collide with them sooner than a small near object that actually arrived sooner. This shows that people partly relied on the heuristic (and in this case misleading) cue of relative size instead of reliable tau information [16,35–37]. In our prior studies [34,37], heuristic cues and reliable cues contributed to TTC judgments in visual and auditory modalities, but the relative importance of heuristic cues was much greater in the auditory modality than in the visual modality. For auditory TTC judgments, we observed a strong reliance on the sound level at the moment when the TTC judgment was made, compatible with an “intensity-arrival effect:” At the same actual TTC, a louder vehicle was judged to arrive earlier than a softer vehicle [34,37,38]. The implication is that measuring the relative contributions of vision and audition to collision judgments is too coarse and incomplete. One must measure the relative contributions of the different cues within the visual modality and the different cues within the auditory modality, particularly because it cannot be assumed that individuals will use the most accurate cues within each modality. The current study measured the relative weights of reliable and heuristic cues within the visual and auditory modalities in TTC judgments, and assessed whether these weights differ between individuals with normal and impaired vision. Due to reliance on peripheral vision and the demands exacted by vision loss, we expected that TTC judgments would show a stronger association with heuristics than more reliably accurate information in individuals with central vision loss.

Our work goes beyond earlier studies in several ways. First, we measured TTC estimates which is a critical component of collision avoidance (our measures of street-crossing decisions will be submitted in a separate manuscript), and we used the well-established prediction-motion task to measure those estimates. Second, we used rigorous reverse-correlation techniques to quantitatively measure the relative importance of different types of auditory and visual cues, including both heuristic cues and reliable cues. Third, we focused our study on people who had AMD in both eyes. Finally, we analyzed the data with traditional comparisons of impaired vision vs. normal vision and also with comparisons of two subgroups of impaired vision that differed in severity of disease.

### Objectives

Our first objective was to determine whether individuals who have partial vision loss compensate by relying on their hearing while making TTC judgments when both visual and auditory cues are available. Our second objective was to determine whether individuals with partial vision loss achieve better collision judgments when they have auditory information in addition to their residual vision, compared to residual vision alone, and how performance differs from individuals with normal vision. Our third objective was to measure how much weight individuals with partial vision loss assign to reliably accurate information compared to less reliable heuristics during collision judgments, and to compare these relative weights to individuals with normal vision.

To achieve our goals, we used state-of-the-art virtual reality (VR) technologies that simulated moving vehicles in a 3D environment in both the visual and auditory modalities. Participants saw and heard an approaching vehicle from the perspective of a pedestrian located at the curb. The vehicle disappeared and participants estimated when they thought the vehicle would reach them. In three separate modality conditions, simulations were presented in the visual modality, the auditory modality, or both modalities concurrently. In our main analysis, we estimated relative cue weights using a psychophysical reverse-correlation approach described in detail in our previous papers on TTC estimation [34,37]. We also analyzed mean estimated TTCs and their intraindividual variability. We hypothesized that (a) although vision is impaired, individuals with central vision loss would rely at least partially on their residual vision rather than solely on auditory information. (b) Individuals with central vision loss (and thus less effective use of looming and binocular vision, greater attentional demands, and degraded ability to integrate visual and auditory cues [39,40], would rely more on heuristic visual and auditory cues than reliable visual and auditory cues, and this difference would be greater compared to individuals comparable in age with normal vision. (c) Individuals with central vision loss would exhibit less accurate and less precise TTC estimates in the V condition compared to individuals with normal vision, potentially a source of collisions with approaching objects. We also expected that performance differences between groups with central vision loss and normal vision would be greater for those with more severe loss compared to moderate loss.

## Method

### Participants

A total of 50 older adults participated in the study; 25 had wet or dry age-related macular degeneration (AMD) in both eyes, and 25 (the control group) did not have AMD and had normal vision (defined as 20/40 or better and meeting criteria for having a driver’s license). We refer to these groups as the Impaired Vision Cohort (IV) and Normal Vision Cohort (NV), respectively. Mean age was 78.1 and 68.4 years, respectively. They differed significantly in age, *t*(48) = 4.70, *p* < .001, but not in the score on the blind (telephone) version of the Montreal Cognitive Assessment (T-MoCA, [41,42], *t*(48) = 0.60, *p* = .55). See below for a descrip*t*ion of the cognitive test. Participants were recruited primarily from a retina care center. The study also was advertised through radio announcements, flyers, listservs, emails, senior citizen centers, churches, alumni organizations, Craigslist, and word of mouth. Characteristics of participants who completed the study are shown in Table 1. In some of the analyses, several participants were excluded from certain conditions due to technical and other issues; this is described in the Results Section.

**Table 1:** Characteristics of all participants who completed the study^a^.

	Impaired Vision Cohort (n = 25)			Normal Vision Cohort (n = 25)		
	Mean	Median	Range	Mean	Median	Range
**Age, years**	78.1	79	59-91	68.4	68	60-88
**Male, number**	9			8		
**T-MoCA**	19.32	20	14-22	19.64	20	15-22
**Walking speed m/s**	0.997	1.012	0.44-1.45	1.179	1.171	0.85-1.82
**Right Eye Visual Acuity LogMar**	0.57	0.48	−0.10 to 1.62	0.0384	0.02	−0.16 to.040
**Left Eye Visual Acuity LogMar**	0.371	0.34	−0.06 to 1.12	0.0056	0	−0.16 to 0.28
**Better Eye Visual Acuity** **LogMar**	0.2696	0.24	−0.1 to 0.98	−0.0264	−0.02	−0.16 to 0.16
**Right Eye Log Contrast Sensitivity**	1.074	1.05	0-1.65	1.524	1.65	1.05-1.65
**Left Eye Log Contrast Sensitivity**	1.11	1.35	0.15-1.65	1.53	1.65	1.2-1.65
**Titmus Stereo Test (arcsec)**	356	300	60-800	118.5	80	40-400

^a^ **(One IV participant was excluded from all analyses).**

All participants completed a comprehensive ophthalmology exam which included measures of visual acuity (Early Treatment Diabetic Retinopathy Study; ETDRS), contrast sensitivity (Pelli-Robson), visual field and central scotomas (Humphrey Visual Field Analyzer 24−2), and non-invasive imaging techniques of fundus autofluorescence (FAF) and spectral-domain optical coherence tomography (SD-OCT). A summary of results is shown in Table 2. We excluded participants if they had impaired vision in one eye and normal vision in the other eye, if they had only light perception in either eye, or if they had color vision deficiencies or other ocular disorders in conjunction with their AMD (glaucoma, retinal detachment, or any other major ocular disorders) that would interfere with study participation.

**Table 2:** Ophthalmological characteristics of impaired vision cohort who completed the study^a^.

Age of Disease Onset, Years					
	Mean	Median	Range		
Right Eye	72.96	72	55-84		
Left Eye	72.70	72	55-84		
**Severity and Stage of Disease: Number and Percentage of Participants**					
**Left Eye Severity**	**N**	**%**	**Right Eye Severity**	**N**	**%**
Nonexudative	10	40	Nonexudative	12	48
Exudative	12	48	Exudative	10	40
Exudative, Nonexudative	3	12	Exudative, Nonexudative	3	12
**Left Eye Stage**			**Right Eye Stage**		
Advanced atrophic with subfoveal involvement	3	12	Advanced atrophic with subfoveal involvement	5	20
Advanced atrophic without subfoveal involvement	2	8	Advanced atrophic without subfoveal involvement	1	4
With inactive choroidal neovascularization	10	40	With inactive choroidal neovascularization	8	32
With inactive choroidal neovascularization, Advanced atrophic without subfoveal involvement	1	4	With inactive choroidal neovascularization, Advanced atrophic without subfoveal involvement	1	4
With active choroidal neovascularization	2	8	With active choroidal neovascularization	2	8
Early Dry Stage	2	8	Early Dry Stage	2	8
Intermediate Dry Stage	3	12	Intermediate Dry Stage	4	16
With active choroidal neovascularization, Advanced atrophic with subfoveal involvement	1	4	With active choroidal neovascularization, Advanced atrophic with subfoveal involvement	1	4
With active choroidal neovascularization, Advanced atrophic without subfoveal involvement	1	4	With active choroidal neovascularization, Advanced atrophic without subfoveal involvement	1	4
**Extent of Anatomic Damage/ Macular Involvement from OCT Images**					
**Left Eye**			**Right Eye**		
0 = none	4	16	0 = none	4	16
1 = small	5	20	1 = small^b^	8	32
2 = medium	6	24	2 = medium	4	16
3 = large	7	28	3 = large	3	12
4 = very large	3	12	4 = very large	6	24
**Fovea Status/Damage**					
0= neither eye	7	28			
1 = od right	5	20			
2 = os left	7	28			
3 = ou both	6	24			

^a^One IV participant was excluded from all analyses

^b^There were two people in the NV group who had small involvement

All participants received otoscopic and tympanometric examinations to rule out ear-related medical issues. Audiometric thresholds were obtained with a diagnostic audiometer meeting American National Standards Institute (ANSI) standards for audiometers in S3.6-2018 [43] in an acoustical enclosure that also satisfied its respective ANSI standard [44,45]. Audiometric testing was performed under headphones using pulsed pure tone stimuli for each ear separately. Testing was conducted for octave frequencies between 125–8000 Hz for air conduction hearing thresholds. For individuals with hearing loss (i.e., audiometric thresholds > 25 decibels [dB] hearing level [HL]) and who had access to bilateral hearing aids, soundfield (non-ear-specific), audiometric threshold testing was conducted in the bilateral-aided condition at 0° azimuth and 1 m speaker-to-ear distance. We used warbled pure tone stimuli to determine functional hearing threshold levels with hearing aids active and in-situ for octave frequencies 500–4000 Hz. To ensure the audibility of the vehicle sound in the TTC estimation task, participants were included in the study if they had normal hearing (≤ 25 dB HL) or mild hearing loss (≤ 40 dB HL) at 500 Hz and 1000 Hz (aided or unaided). Detection of higher frequencies was less critical because the sounds of the stimuli (vehicles) fell mostly in the low frequency range (< 2000 Hz) [38]. Participants were excluded if they were cochlear implant users or had medical otologic problems within the last five years including sudden hearing loss, pain or pressure in the ears, or otorrhea.

To compute pure tone average (PTA) hearing levels in the unaided participants, we first identified the better-ear air conduction thresholds, separately at 500, 1000, and 2000 Hz. These three frequency-specific better-ear hearing levels were then averaged. For those with hearing loss using hearing aids (aided group), PTAs were calculated using the average hearing level across the test frequencies of 500, 1000, and 2000 Hz for thresholds obtained in the bilateral, aided soundfield condition previously described. The interaural asymmetry of hearing levels in the same frequency range was computed as the arithmetic mean of the absolute values of the difference in air conduction HLs between the left and right ears at 500, 1000, and 2000 Hz. Mean PTA interaural asymmetries were small and not clinically significant [46].

A summary of participants’ audiometric results by vision group (IV and NV) is provided in Table 3. The mean PTA was significantly higher (worse) for the IV group compared to the NV group, *t*(46.85) = 2.085, *p* = .042. The average asymmetry of the hearing thresholds was also significantly higher in the IV compared to NV group, *t*(41.63) = 2.369, p = .023. Both the IV and NV groups had overall mean PTAs (unaided or aided) of audiometric thresholds at 500, 1000, and 2000 Hz that were within a normal hearing range of <= 25 dB HL (NV overall mean PTA = 15.47 dB HL [*SD* = 7.78]; IV overall mean PTA = 19.73 dB HL [*SD* = 6.64]). Descriptive audiometric results indicated normal or near-normal average hearing levels that have low risk of confounding safe crossing judgments among participants, given that acoustic stimuli were presented suprathreshold.

**Table 3:** Audiometric results for impaired vision (IV) and normal vision (NV) cohorts.

	IV			NV		
	IV Group (overall)	Unaided Group	Aided Group	NV Group (overall)	Unaided Group	Aided Group
**N**	25	14	11	25	24	1
**Mean PTA**^a^ (*SD***) in dB HL**^b^	19.73 (6.64)	18.21 (6.08)	21.67 (7.11)	15.47 (7.78)	14.93 (7.46)	28.33 (n/a)^c^
**Median PTA in dB HL**	21.67	18.33	23.33	13.33	13.33	28.33
**PTA range in dB HL**	6.67–30.00	6.67–28.33	8.33–30.00	1.67–33.33	1.67–33.33	28.33–28.33
**Mean Interaural Asymmetry for PTA (** *SD* **) in dB HL**	4.60 (2.82)	3.81 (2.57)	5.61 (2.91)	3.00 (1.86)	2.92 (1.86)	5.00 (n/a)^c^

^a^PTA calculations for the normal hearing group used the average of the better air conduction thresholds at each frequency under headphones between the left and right ears. PTA for the aided group used thresholds obtained in the bilateral, aided soundfield condition. One IV participant was excluded from all analyses because they did not follow instructions for the TTC task.

^b^dB HL = decibels hearing level as measured on the audiometer; PTA = pure tone average of hearing thresholds at 500, 1000, and 2000 Hz.

^c^Indicates that standard deviation was not reported due to subgroup sample size of *N* = 1.

Individuals were not eligible for the study if they reported having a loss of mobility that necessitated assistance by a normal-sighted adult or service animal, or use of a wheelchair; a seizure disorder or other illnesses activated by viewing moving displays or displays with fast-flicker or virtual reality displays; Parkinson’s Disease or any other impairments in motor behavior that would interfere with study participation; inability to wear a virtual reality headset; or Alzheimer’s Disease or other forms of dementia. Participants were given the Montreal Cognitive Assessment (T-MoCA- BLIND; [41,42]). Stereoscopic acuity was measured with a Titmus Test [47], presented in the virtual reality headset. Nine binocular disparities (800, 400, 200, 140, 100, 80, 60, 50 and 40 s of arc) were presented. Mean results are shown in Table 1.

Participants were compensated at a rate between $25 and $75 (depending on duration) per completed session up to a total of $300, with additional funds for parking/transportation fees. We arranged and paid for transportation to and from the study/clinic sites if requested by the participant. This research complied with the American Psychological Association Code of Ethics and was approved by the Institutional Review Board at Rice University (IRB-FY2021–25). Written informed consent was obtained from each participant. Recruitment began on approximately January 13, 2022, and ended on approximately March 27, 2024.

### Visual simulation of approaching vehicles

Visual simulations of approaching vehicles were created with a modified version of previously developed in-house software programmed in the Unity3D real-time development platform [48,49]. The in-house code was extended to integrate TASCAR (see below) for synchronous sound generation and modified to meet experimental parameters for timing, object dimensions, and scene layout. The Unity script generated the motion of the visually presented vehicles, triggered the audio, controlled the sequence of trials, and recorded the responses of participants. The Unity game engine rendered interactive stereoscopic simulations of the scene using head tracking. Audio simulations of approaching vehicles were created with an interactive audiovisual virtual-reality (VR) system based on a setup created at the Johannes Gutenberg University Mainz (see details in [38]) that creates physically plausible acoustic 3D simulations of vehicles moving in depth toward the participant. The combined audio plus visual simulation system included two Dell Optiplex computers (10-core i9-10900K processor; 32GB RAM) with NVIDIA GeForce RTX 2070 Super video cards. One computer ran Windows 10 and the other ran Ubuntu 20.04. The Windows computer generated the visual displays with Unity 19.4.9. The Ubuntu computer generated the auditory simulations in TASCAR (Toolbox for Acoustic Scene Creation and Rendering; [50]; http://www.tascar.org/).

**Fig 1:**
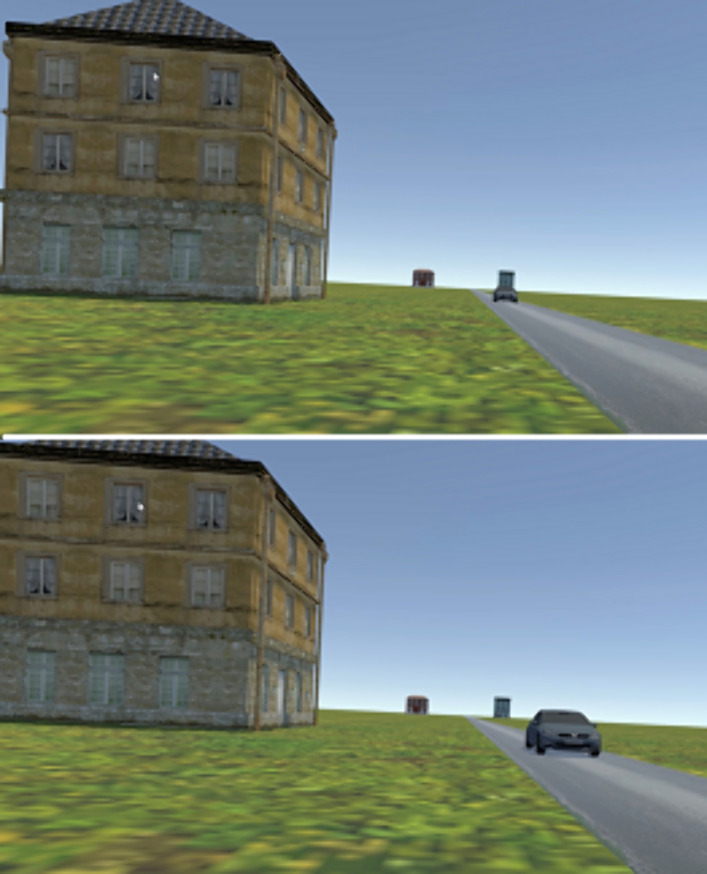
Screenshots of approximate first video frame of approaching car (top panel) and last video frame prior to car’s disappearance (“occlusion”; bottom panel).

The simulated traffic scene consisted of a flat rural landscape with a vehicle moving on a straight single-lane road surrounded by several buildings, sky, and grass (Fig 1). The road was 3.65 m wide (12 feet), a typical width in the USA. The vehicle was either a (small) car (1.77 m × 1.41 m × 4.11 m: width × height × length) or a (large) truck (2.032 m × 2.08 m x 5.89 m). In both visual and auditory displays, the vehicle approached the participant who viewed the scene and listened to the sound of the approaching vehicle from the perspective of a pedestrian positioned at a distance of 1 m from the edge of the road, as a pedestrian intending to cross the street would do normally. The vehicle approached for 3 s and then it disappeared (i.e., it was no longer visible or audible). The visual stimuli were presented stereoscopically with Steam VR on an HTC Vive Pro Eye VR headset with 110 deg field of view, 1440 × 1600 pixels per eye and 90 Hz refresh rate. Due to the head-tracking of the virtual reality headset, participants were able to explore the scene with head movements.

### Auditory simulation of approaching vehicles

The auditory simulation used a source-based approach. As explained in detail in [38], audio recordings of a real vehicle were used as sound sources in the acoustic simulation software TASCAR to produce a physically plausible spatial auditory simulation of approaching vehicles in the experimental setting. The source signals were recordings of a gasoline-powered Kia Rio 1.0 T-GDI 120 (2019, 1.0 l, 88 kW, 3 cylinders) with manual transmission and Continental summer tires (ContiSportContact 5, 205/45 R17) driving on a test track with a dry asphalt surface at various constant speeds (for details see [38]). Four free-field microphones (Roga MI-17), mounted to the chassis of the car (one above each of the front axles, one above the right rear axle, and one centrally on the engine hood), recorded the vehicle sounds during the drives. Synchronously, the GPS position of the car was recorded at high precision such that at each time point in the audio signals, the position, speed, and acceleration of the vehicle was known. In the simulations, the four microphone signals were assigned to four point sources in TASCAR. The motions of the sound sources in space were simulated in TASCAR, which provides a physically plausible interactive simulation of the dynamic spatial sound field, with dynamic processing of the geometry of the acoustic scene and acoustic modeling of the sound transmission from the sources to the receiver. TASCAR renders the scene using sound field synthesis. As the vehicle moved through the acoustic scene, TASCAR dynamically updated the position of the vehicle and modelled the directionality of sound sources, the distance-dependent change in sound level caused by spherical spreading and air absorption, the distance-dependent sound propagation time (which can lead to Doppler effects, for example), and sound reflections from the ground and other surfaces, using a first-order image sound source method [51]. This simulation approach created realistic vehicle sounds and provided all relevant monaural and binaural distance and motion cues, such as dynamic changes in intensity, interaural time and level differences, and frequency spectrum.

The geometry of the simulated auditory scene was identical to the visual scene. The surfaces contained in the scene were simulated with plausible acoustic reflection properties, based on ISO 9613–2:1999-10 [52]. The reflectance of the road surface and the house fronts was set, respectively, to ρ = *I*_*r*_/*I*_*0*_* *= 1.0 and ρ = 0.8, where *I*_*r*_ is the acoustic intensity of the reflected sound wave and *I*_*0*_ is the intensity of the incoming wave. The reflectance of the grass surfaces was set to ρ = 0.5. The sound reflections were modeled with a first-order IIR low pass filter with a cut-off frequency of 5 kHz. The “scattering” parameter in TASCAR was set to 0.5 for all surfaces.

For the reproduction of the spatial sound field, we used sound field synthesis (e.g., [53]), rendering in 2D 7^th^-order Ambisonics with max-*r*_E_ decoding [54,55]. The auditory stimuli were played back on 16 Genelec 8020 loudspeakers configured in a circular array with a 5-m diameter, positioned at approximately ear height. The array was installed in a large laboratory space (approximately 9.47 m long × 7.31 m wide × 2.74 m high). The 16 loudspeakers of the array were controlled by an audio converter (Ferrofish Pulse 16, 24 bit audio resolution, *f*_s_ = 44.1 kHz), which received the audio signals from an RME HDSPe RayDAT audio card in the Linux computer running TASCAR. Acoustic calibration and smoothing of the frequency responses of the loudspeaker array was performed using the TASCAR Speaker Calibration Tool and a class-1 sound level meter (Larson Davis LxT1 with PCB Model 377B02 free field condenser microphone) placed in the center of the loudspeaker array and at the participant’s ear height. During calibration, level differences between the 16 loudspeakers were compensated and the sound pressure levels of a point source and a diffuse sound field were calibrated. The sound levels from the calibrated microphones mounted on the cars during the vehicle recordings on the test track were used to set the sound levels of the simulated sound sources.

On each trial, we selected a random time interval corresponding to the required presentation duration of 3 s from the available recording duration (20 s or more). Thus, the presented sound of the car differed slightly from trial to trial for each presented velocity, as the sound of real vehicles would do, increasing the ecological validity. The auditory simulation in TASCAR was started synchronously with the motion of the visual object in the visual simulation.

To reduce interference from acoustic reflections, the laboratory area was sound-treated. A layer of Techlite acoustic panels made from Basotect G+ acoustic foam from BASF was attached to the walls (10 cm thick; absorption coefficient of at least α = 0.9 at frequencies above 400 Hz) and ceiling (4.4 cm thick; absorption coefficient of at least α = .6 at frequencies above 400 Hz). To reduce reflections from the floor, a thick carpet was placed inside the entire room.

In addition to the simulated approaching vehicle, a first-order Ambisonics recording from a quiet residential area containing remote traffic sound and sounds of birds etc. [56] was presented as background noise (*L*_*Aeq*_ = 50.0 dBA). The level of the background noise was selected so that it masked the ambient noise (e.g., air conditioner) in the laboratory space. The ambient sound level of 50 dBA is similar to what was reported by [57] for intersections in a residential area in the US.

### Procedure

All participants completed a time-to-collision estimation (TTC) task and a street-crossing task. This paper discusses only the TTC task. We measured how long it took them to walk a distance in the hallway that matched the width of the virtual road for use in the street-crossing task. The walking task was followed by surveys that characterized hearing with the Speech, Spatial, and Qualities of Hearing scale (SSQ) [58]- the spatial subscale of the SSQ, and a short form of the SSQ (15iSSQ) that contained all three subscales of speech, spatial, and qualities [59].

#### Time-to-collision estimation task (TTC task).

In three *modality conditions*, the approaching vehicle was presented only visually (V), only auditorily (A), or audio-visually (AV). Participants experienced the displays while seated in the center of the speaker array. On each trial, a single vehicle approached the participant at a constant speed for 3.0 s and then disappeared (i.e., it was no longer visible and/or audible, as if masked by an invisible occluder). We measured participants’ estimates of the time the vehicle would reach them, that is, the vehicle’s time-to-collision (TTC) with a prediction-motion task often used in prior studies [17,60–62]. Specifically, participants were instructed to press a button on the Vive controller at the exact time when they thought that the vehicle would reach them, had the object continued to move with the same velocity after it was no longer visible/audible (“occluded”). TTC judgments were measured as the time between the last video frame or audio sample of the presentation and the time when the participant pressed the button.

The vehicle’s TTC at occlusion (1.125–5.7 s) and velocity (20 km/h – 60 km/h) were varied. In addition, the size of the visually presented vehicle (small car, large truck) and the source intensity of the auditorily presented vehicle (lower versus higher source intensity, separated in sound level by 15 dB) were varied. A major objective of the experiment was to measure the relative weighting of auditory and visual cues. As explained in our previous work [34,37], estimation of relative weights is only possible for cues that are not perfectly correlated. For this reason, in the AV condition, the TTC at occlusion and the velocity of the visually simulated vehicle were “jittered” slightly against the TTC and velocity of the auditorily simulated vehicle on a subset of trials. The primary values of the TTC at occlusion of the approaching vehicle were 1.5 s, 3.25 s, and 5.0 s. At each of the three primary TTC levels, a “jitter” of 0.375 s, 0.5 s, and 0.7 s, respectively, was introduced to create 9 TTC values ranging from 1.125 s to 5.7 s. For instance, at the TTC level of 1.5 s, the jitter value was 0.375 s resulting in TTC values of 1.125 s (1.5 s − 0.375 s) and 1.875 s (1.5 s + 0.375 s). These TTC values were used to create 9 different combinations of auditory TTC and visual TTC at occlusion representing conditions in which the TTC was the same for A and V, TTC was longer for A or TTC was longer for V. Refer to Table 4.

**Table 4:** Combinations of auditory time-to-collision (TTC_A_) and visual TTC (TTC_v_) when the object was occluded in the audio-visual condition, based on varying the auditory and visual TTC around three TTC levels. The same TTCs as in columns TTC_A_ and TTC_V_ were presented in the single-modality conditions (A and V, respectively).

TTC level (s)	TTC_V_ (s)	TTC_A_ (s)
1.5	1.125	1.875
	1.5	1.5
	1.875	1.125
3.25	2.75	3.75
	3.25	3.25
	3.75	2.75
5.0	4.3	5.7
	5	5
	5.7	4.3

Because the acoustic vehicle recordings were only available at 6 different constant velocities (10 km/h to 60 km/h in steps of 10 km/h), the velocities presented in the experiment were taken from this set of velocities. The primary velocities of the vehicle were 30 km/h and 50 km/h. At each of these two velocity levels, a jitter of 10 km/h was introduced to create 5 velocities ranging from 20 km/h to 60 km/h. In the AV condition, these velocity values were used to create 10 different combinations of auditory velocity and visual velocity representing trials in which the velocity was the same for A and V, velocity was faster for A or velocity was faster for V. Refer to Table 5.

**Table 5:** Combinations of auditory velocity (*v*_A_) and visual velocity (*v*_V_) in the audio-visual condition, based on varying the auditory and visual velocity around two velocity levels. The same velocity values as in columns *v*_A_ and *v*_V_ were presented in the single-modality conditions (A and V, respectively).

Velocity level (km/h)	*v*_v_ (km/h)	*v*_A_ (km/h)
30	20	30
	30	20
	30	30
	30	40
	40	30
50	40	50
	50	40
	50	50
	50	60
	60	50

The size of the visual object was varied. The vehicle was either a (small) car (1.77 m × 1.41 m × 4.11 m: width × height × length) or a (large) truck (2.032 m × 2.08 m × 5.89 m). The vehicle source intensity was varied by 15 dB, independently of the other factors, creating a high and a low source intensity, by presenting the vehicle sounds with an audio gain of +17.5 dB and +2.5 dB, respectively, to the original vehicle recordings. The first two participants tested received source intensities that were 10 dB lower (i.e., audio gains of +7.5 dB and –7.5 dB). Because that resulted in problems with the audibility of the car sounds, the source intensities were increased to the values above for all other participants. In the AV condition, the 9 combinations of visual TTC and auditory TTC were factorially crossed with the 10 combinations of visual velocity and auditory velocity, and the two values each of vehicle size and source intensity. This resulted in 360 unique experimental conditions which were presented once to each participant in four separate blocks of 90 trials. The complete realization of the AV condition took about 45 minutes.

In the single-modality conditions (A and V), the same values of TTC at occlusion and velocity as in the AV condition were presented. In the V condition, the factorial combination of 9 visual TTCs, 5 visual velocities, and two vehicle sizes resulted in 90 unique conditions presented once to each participant during one block that took about 15 minutes. In the A condition, the factorial combination of 9 auditory TTCs and 5 auditory velocities and two source intensities resulted in 90 unique conditions presented once to each participant during one block that took about 15 minutes. Participants were given 9 practice trials, 3 in each of the modality conditions, to get familiarized with the task. Except for the manipulations described, the characteristics of the road, scenery, and vehicle remained the same across trials.

#### Sessions.

Each participant completed six sessions. In the first session, they completed the consenting process, and the T-MoCA (Montreal Cognitive Assessment BLIND v 8.1; [41,42]). In the second and third sessions, they completed the hearing and vision exams, respectively, as detailed previously. In the fourth, fifth, and sixth sessions, they completed the TTC estimation task (and street-crossing task- not reported here, with order of task counterbalanced across participants). In session 4, they also completed the i15SSQ, and the Titmus (stereovision) test in the VR headset. In the sixth session they completed the spatial subscale of the SSQ. The order of modality conditions (A, V, AV) was counterbalanced. Within each block, trials were presented in randomized order. The fourth, fifth, and sixth sessions took about 2 hours each. The sessions were too long for participants to stand; they completed the task while seated in a chair.

## Results

We performed different analyses for different purposes. In our main analysis, we estimated relative cue weights using a psychophysical reverse-correlation approach described in detail in our previous papers on TTC estimation [34,37]. In this type of perceptual weight analysis, which stands in the tradition of “molecular psychophysics” first introduced by [63], the trial-by-trial data are analyzed to identify how the response (i.e., the estimated TTC) depends on a range of potential cues, and which of these cues are most important for the judgment (e.g., [34,64–66]).

In a second step, we analyzed the mean estimated TTCs and their intraindividual variability (standard deviation; *SD*), to identify differences between the IV and NV group and effects of modality condition, TTC at occlusion, velocity, vehicle size, and acoustic source intensity on these dependent variables. We begin with the analysis of cue weights.

### Reverse-correlation analyses of relative contributions of visual and auditory cues

Our analyses of cues weights and their relative importance were similar to those we reported previously [34,37]. We assessed the relative contributions of the different potential cues within and across modalities by using psychophysical reverse correlation.

#### Selection of visual and auditory cues.

**Fig 2:**
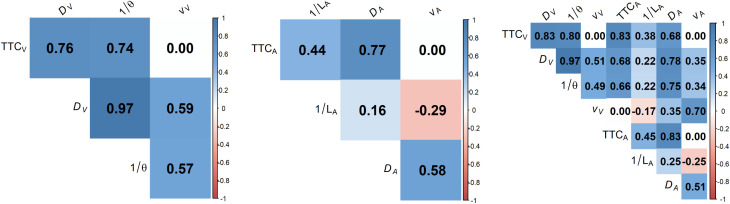
Pearson correlations between predictors, per modality condition. The left, middle, and right panel shows the V (visual only), A (auditory only), and AV (audiovisual) condition, respectively.

For an ideal observer making perfectly accurate TTC estimations, the TTC estimates are completely determined by the presented or actual TTC – the time remaining until the car would reach the pedestrian- at the moment the car disappeared. However, the literature on TTC estimation clearly showed that factors such as the velocity of an approaching object as well as its distance, (optical) size, and sound intensity can affect estimated TTCs (e.g., [34,35,37,38,67]). For this reason, we included a range of potential cues as predictors in the regression analyses that we used to estimate cue weights from the trial-by-trial data. In the V condition, these encompassed the TTC at occlusion (TTC_V_), the distance at occlusion (*D*_*V*_), and the velocity (*v*_*V*_). The optical size (ϴ, defined as the angle subtended by an imaginary diagonal line across the car’s front, as seen from the participant’s position) at the moment of occlusion was computed. We included the reciprocal of the optical size at occlusion, 1/ϴ, as a predictor, so that this predictor was positively related to the presented TTC, just as the other predictors. In the A condition, the predictors again included the TTC and distance at occlusion, and the velocity (TTC_A_, *D*_*A*_, *v*_*A*_). The reciprocal of the A-weighted vehicle sound level at occlusion (1/*L*_*A*_) also was included. In the AV condition, the sets of visual and auditory predictors were combined. Note that although our experimental design ensured that none of the predictors were perfectly correlated (which would have made the estimation of cue weights impossible), most of these cues were correlated with each other (Fig 2), which is generally unavoidable in TTC experiments. A particularly high correlation was present for the visual cues *D*_*v*_ and 1/ϴ. Other potential cues, such as the rate of change in optical size, were so highly correlated with some of the already included predictors that it was not possible to add them.

#### Estimation of cue weights and assessment of relative variable importance.

Using the psychophysical reverse-correlation approach, our aim was to identify cues that were associated with the estimated TTC, and to gauge their relative importance. Because there is not a single, “gold standard” approach for analyzing and comparing the relative importance of several correlated predictors, we used two different, complementary types of regression analyses which approach the bias-variance trade-off (for an excellent introduction see chapter 2.2 in [68]) from slightly different angles (see also [69]), dominance analysis [70], and variable selection based on cross-validation using the Lasso [71].

In the first set of analyses, which was identical to the type of analyses we had used in two previous papers [34,37], ordinary least squares (OLS) multiple regression models containing the full sets of predictors as listed above were fitted to the individual data, separately per modality condition, and the relative importance of the predictors was assessed using general dominance weights (GDW; [70]). The “dominance analysis” approach proposed by [72] accounts for the fact that the predictors were partly correlated as discussed above, and thus the relative importance of the predictors could not be gauged by simply considering the squared standardized regression coefficients (cf. [73]). Dominance analysis provides a quantitative measure of relative importance by examining the change in the explained variance (Δ*R*^2^) resulting from adding a given predictor to all possible regression models containing subsets of the predictors. In this approach, a predictor’s *general dominance weight* (GDW; [70]) is defined as its mean squared semi-partial correlation across all possible regression models containing subsets of the predictors. The GDW indexes a variable’s contribution to the prediction of the dependent variable, by itself and in combination with the other predictors. GDWs have several desirable properties for quantifying variance importance in a regression model (e.g., [73,74]).

As mentioned above, data from one participant in the A and AV conditions were excluded from the analyses due to inaudibility. In addition, one participant responded at virtually the same time after occlusion on every trial – indicating problems understanding the TTC estimation task, and was therefore completely excluded from the analysis. As a result, 25 participants from the NV group entered the analysis in all three modality conditions. For the IV group, data from 24 participants were analyzed in the V condition, and data from 23 participants in the A and AV conditions. We excluded trials in which a technical problem had occurred, that is, when the sound of the vehicle did not play (due to technical issues), or when the participant pressed the advance button too soon causing a trial to be skipped (due to technical issues). We also excluded trials in which participants responded before the vehicle disappeared in the V or AV conditions (resulting in negative estimated TTCs) because during such early responses there was still visual information to indicate that the car was still at some distance from their position. We did not exclude such early responses in the A condition because the distance between participant and vehicle was less obvious when no visual information was available. Thus, it was plausible that participants might on some trials judge the car to arrive at their position even when the vehicle sound was still being presented and the vehicle was – in fact − still at some distance from them.

Separate multiple regression models were fitted, for each combination of participant and modality condition, to the set of data that remained after trials with technical errors and negative estimated TTCs in the V and AV condition had been excluded (as described previously). All variables were *z*-standardized. The predictors were entered simultaneously (additive model). We performed regression diagnostics by analyzing the DFFITS index proposed by [75], which measures the influence of an observation on the fitted model in terms of the change in fit when the observation is deleted, and studentized residuals. Per fitted model, observations with an “outlying” DFFITS value more than three inter-quartile ranges below the first or above the third quantile, or an absolute value of the studentized residual of greater than 2.0 [75] were excluded, and the regression model was fitted again. Because for some participants a few “influential” trials remained after this step, we included a second iteration where trials in the latter regression model with DFFITS values more than 3 inter-quartile ranges below the first or above the third quantile and an absolute value of the studentized residual of greater than 2.0 were excluded, and the regression model was fitted a third time. Across participants, these two iterations of detection of influential data points resulted in the exclusion of between 1 and 11 (*Mdn* = 5) of the up to 90 trials collected in the A condition, between 2 and 11 (*Mdn* = 6) of the up to 90 trials in the V condition, and between 3 and 30 (*Mdn *= 13) of the up to 360 trials in the AV condition. In our experimental design, in the A, V, and AV conditions the maximum condition index [75] was 8.03, 12.9, and 21.0, respectively. In [75] it was suggested that only condition indices of at least 30 indicate potential problems with multicollinearity.

**Fig 3:**
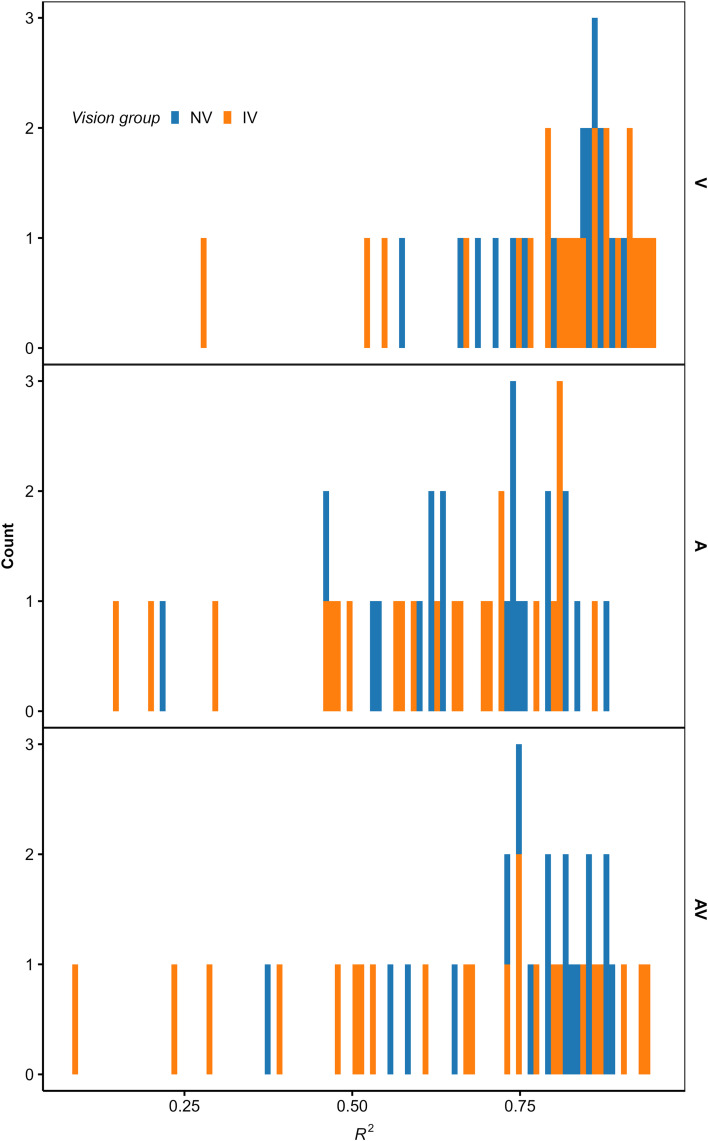
Histogram of the coefficient of determination (*R*^2^) for the fitted OLS regression models, per modality condition. Blue: NV group. Orange: IV group. The top, middle, and bottom panel shows the V, A, and AV condition, respectively.

Across the 145 fitted models, *R*^2^ ranged from 0.085 to 0.947 (*Mdn* = 0.768). As shown in Fig 3, for most fitted models, the goodness of fit was satisfactory (i.e., *R*^2^ > 0.5). This indicates that although the TTC estimations were not unbiased (Figs 11–13), most participants responded rather consistently (i.e., the level of “internal noise” was not overly high), and the analyzed cues (i.e., predictors in the regression models) were relevant for the TTC estimations. For one participant, *R*^2^ was low in all modality conditions, indicating high “internal noise” or missing relevant predictors. In fact, the estimated TTCs of this participant showed a bimodal distribution in the AV condition. A few other participants showed a low *R*^2^ in some, but not all modality conditions. In the A condition, *R*^2^ was generally lower than in conditions presenting visual information, but still indicated consistent response strategies because most participants showed an *R*^2^ > 0.5 (Fig 3).

**Fig 4:**
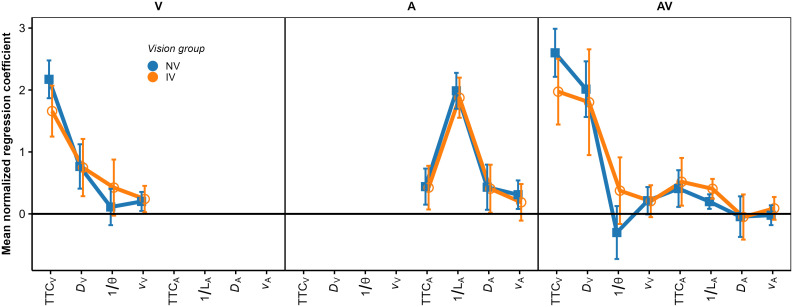
Mean normalized regression coefficients from the OLS regression. The left, middle, and right panel shows the V, A, and AV condition, respectively. Blue squares: NV group. Orange circles: IV group. Error bars show 95% confidence intervals (CIs).

Because the *relative* contributions of the different predictors to the TTC estimation were of interest rather than the absolute magnitude of the regression coefficients, the regression coefficients were normalized for each individual fitted model such that the mean of the absolute values of the three predictors was 1.0. Fig 4 shows the mean normalized regression coefficients.

We structure the report of the results by sensory modality condition. In the *V condition*, all regression coefficients except for 1/ϴ were significantly positive across participants in both IV and NV groups, as shown by the confidence intervals in Fig 4. Thus, compatible with previous results [34,37], the estimated TTC was not completely determined by the presented TTC. Estimated TTC was additionally associated with the distance at occlusion, with longer distances corresponding to longer estimated TTCs. On average, the optical size at occlusion was not significantly associated with the estimated TTC. The regression coefficient for *v*_*V*_ was significantly positive; we have no immediate explanation for this result. To test if the patterns of normalized regression coefficients differed between the NV and the IV group, we conducted a repeated-measures ANOVA (rmANOVA), using a univariate approach with Huynh-Feldt correction for the degrees of freedom [76]. The correction factor $\tilde{\varepsilon }$ is reported, and partial η^2^ is reported as measure of association strength. An α-level of.05 was used for all analyses. Predictor was included as a within-subjects factor and vision group as the between-subjects factor. It showed a significant effect of predictor, *F*(3, 141) = 35.30, $\tilde{\varepsilon }$ = 0.78, *p < *.001, $\eta_{p}^{\text{2}}$ = 0.43. The predictor × vision group interaction was not significant, *F*(3, 141) = 1.68, $\tilde{\varepsilon }$ = 0.78, *p = *0.186. Thus, the patterns of normalized regression coefficients did not differ significantly between the two vision groups.

**Fig 5:**
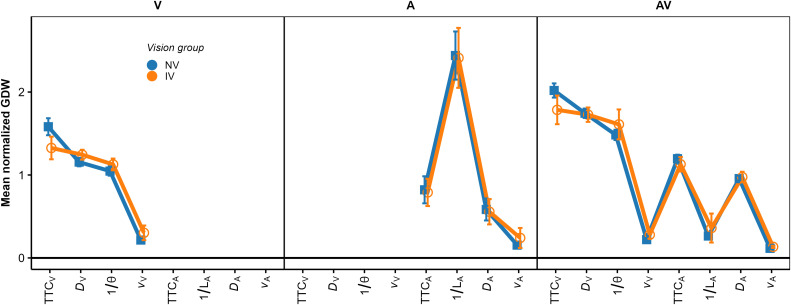
Mean normalized general dominance weights (GDWs). Same format as Fig 4.

The GDWs (our first measure of variable importance; [70]) were normalized to a mean of 1.0 per participant and modality condition, because we were again interested in the patterns of relative GDWs rather than in their absolute magnitudes. The mean normalized GDWs are shown in Fig 5.

In the V condition (left panel), the mean GDW was highest for TTC_V_ in both groups. Thus, in terms of the GDW, the presented TTC was the most important predictor of the estimated TTC. However, particularly for the IV group, the GDW for *D*_V_ was also rather high and not much smaller than the GDW for TTC_V_. The same applied to the GDW for 1/ϴ, although the mean regression coefficient for this predictor was not significantly different from 0. This highlights that the GDWs are not simply equivalent to the squared normalized regression coefficients. An rmANOVA on the normalized GDWs showed a significant effect of predictor, *F*(3, 141) = 271.03, $\tilde{\varepsilon }$ = 0.55, *p < *.001, $\eta_{p}^{\text{2}}$ = 0.85. The predictor × vision group interaction was significant, *F*(3, 141) = 7.41, $\tilde{\varepsilon }$ = 0.55, *p = *0.002, $\eta_{p}^{\text{2}}$ = 0.136, albeit with a relatively small effect size. Thus, the relative importance of the predictors as gauged by the GDW differed significantly between groups in the V condition. Pairwise comparisons between GDWs for the NV and the IV group per predictor were computed via Welch two sample *t*-tests not assuming equal variances. The normalized GDW for TTC_V_ was significantly higher in the NV compared to the IV group, whereas the normalized GDW for *D*_V_ was significantly lower in the NV compared to the IV group. All other pairwise comparisons were non-significant. Taken together, compatible with our expectations, the TTC estimation of the IV group showed a higher relative importance of “heuristic” cues compared to TTC_V_ than for the NV group.

In the second approach to gauging the relative importance of the cues, we used variable selection with the *Lasso* [71], based on cross-validation. The Lasso is a regularized regression method that performs *subset selection*, that is, it selects the most important predictors [68], because the Lasso forces the coefficient estimates of “unimportant” predictors to be exactly equal to zero. The method involves a tuning parameter λ to impose an *l*_1_-penalty [77] on the regression model. We used the mean squared prediction error (MSPE) in 10-fold cross-validation for selecting the best model, that is, the optimal value of λ. The Lasso with a value of λ resulting in the smallest mean MSPE shows the best predictive accuracy on average. With respect to the bias-variance trade-off in statistical learning (e.g., [68]), this approach selects a model that has small variance and can thus be expected to generalize well to new datasets, while the OLS multiple regression model containing all predictors minimizes the bias by providing the best linear prediction for the dataset at hand (small bias). Put differently, the two approaches are complementary, and the Lasso analysis focuses on prediction [78]. To further increase the robustness of the results, we repeated the cross-validation-based model selection with the Lasso 20 times per combination of participant and modality condition, each time randomly partitioning the data into 10 subsets for cross validation. For each combination of participant and modality condition, we computed the proportion (*p*_*Lasso*_) of the 20 CV runs on which a given predictor was included in the selected model (i.e., where the regression coefficient estimated by the Lasso was not equal to 0). We used the relaxed Lasso [79], which was suggested to perform favorably compared to the normal Lasso [80], and implemented the analysis using the R function *cv.glmnet (https://glmnet.stanford.edu/)*. Except for the exclusion of trials with technical errors and negative estimated TTCs as explained above, no additional trials were excluded during the analysis because the repeated cross-validation procedure reduces the influence of “outlying” data points.

**Fig 6:**
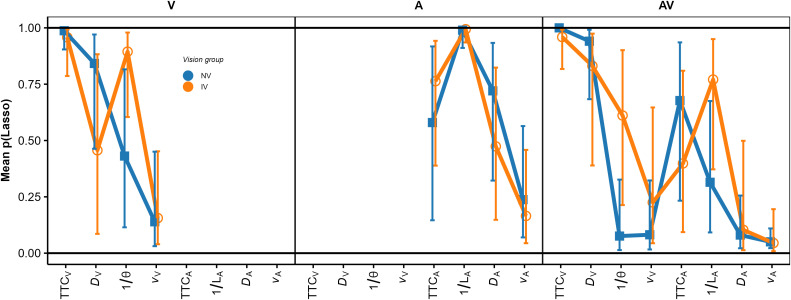
Average values of pLasso per predictor and vision group, plotted separately for each modality condition. Means and 95% confidence intervals were estimated using intercept-only hierarchical logistic regression models with a random intercept (see text).

To aggregate the proportions of CV runs on which a given predictor was selected across participants within each vision group and modality condition, we used intercept-only hierarchical logit models (logistic regression) with a subject-specific random intercept, fitted separately per combination of modality condition, predictor, and vision group. The degrees of freedom were computed according to [81] in SAS PROC GLIMMIX (the SAS syntax is available in OSF; [82]). The goodness of fit was reasonably high, indicated by values of generalized χ^2^ divided by the degrees of freedom of approximately 1.0 (i.e., no indication of overdispersion). Fig 6 shows the estimated mean values of *p*_Lasso_ and their 95% CIs.

In the V condition, the ordering of the average *p*_Lasso_ values for the NV group was identical to the ordering of the GDWs, confirming that TTC_V_ was the most important predictor for this group. For the IV group, *p*_Lasso_ was also highest for TTC_V_, while it was relatively low for *D*_V_ but almost as high for 1/ϴ than for TTC_V_, again indicating a relatively high importance of the optical size at occlusion. This pattern differs from the ordering shown by the GDWs for this vision group (Fig 5), where the GDW for *D*_v_ was similar to the GDW for 1/ϴ. To analyze for a predictor × vision group interaction (i.e., differences between the patterns of *p*_Lasso_ between groups), we used hierarchical logistic regression models with random intercept and slope [83], with a compound-symmetry (CS) covariance structure because with more complex covariance structures, the models did not converge for all modality conditions. The degrees of freedom were again computed according to [81]. For all three modality conditions, the goodness of fit was reasonably high, indicated by values of generalized χ^2^ divided by the degrees of freedom close to 1.0. For the V condition, there was a non-significant pattern consistent with a predictor × group interaction, *F*(3, 145) = 2.49, *p = *.0628. Pairwise comparisons of *p*_Lasso_ between the NV and the IV group per predictor were computed with hierarchical logistic regression models with random intercept (again showing values of generalized χ^2^ divided by the degrees of freedom around 1.0). This showed a significant difference in *p*_Lasso_ between groups for the optical size cue (predictor 1/ϴ), *F*(1, 43.646) = 4.24, *p =* 0.046, for which the mean *p*_Lasso_ was higher for the IV group. For all other predictors, the difference between groups was non-significant.

In the *A modality condition* (middle panel in Fig 4), all regression coefficients except for *v*_*A*_ in the IV group were significantly positive across participants in both the IV and the NV group. Thus, as for the V condition, the estimated TTC was not completely determined by the presented TTC. In particular, it was strongly associated with the inverse of the sound level at occlusion, with higher values of 1/*L*_*A*_ (i.e., lower sound levels at occlusion) corresponding to longer estimated TTCs. This is compatible with the intensity-arrival effect observed in previous studies [34,37,38,84]. The distance at occlusion was also significantly positively associated with the estimated TTC. An rmANOVA with predictor as the within-subjects factor and vision group as the between-subjects factor showed a significant effect of predictor, *F*(3, 138) = 43.71, $\tilde{\varepsilon }$ = 0.63, *p < *.001, $\eta_{p}^{\text{2}}$ = 0.49. The predictor × group interaction was not significant, *F*(3, 138) = 0.06, $\tilde{\varepsilon }$ = 0.63, *p = *0.94. The patterns of normalized GDWs (middle panel in Fig 5) were very similar between the two groups. The mean GDW was highest for 1/*L*_*A*_, compatible with the high reliance on final sound level reported in previous studies on A-only TTC estimation [34,37]. In terms of the GDW, the second-important predictor was TTC_A_, followed by *D*_*A*_. An rmANOVA on the normalized GDWs showed a significant effect of predictor, *F*(3, 138) = 154.91, $\tilde{\varepsilon }$ = 0.39, *p < *.001, $\eta_{p}^{\text{2}}$ = 0.77. The predictor × group interaction was not significant, *F*(3, 138) = 0.13, *p = *0.76. The pattern of variable importance shown by *p*_Lasso_ was largely compatible with the GDWs. As shown in the middle panel in Fig 6, the ordering of the average *p*_Lasso_ values was identical to the ordering of the GDWs for both groups, confirming that 1/*L*_*A*_ was the most important predictor of the estimated TTC. For the IV group, the mean *p*_Lasso_ for TTC_A_ was slightly higher than for the NV group, while on average *p*_Lasso_ for *D*_A_ and *v*_A_ was slightly lower than for the NV group. However, a hierarchical logistic regression model (same variant and parameters as for the V condition) showed no significant predictor × group interaction, *F*(3, 153.45) = 0.706, *p = *.55.

In the *AV modality condition* (right panel in Fig 4), the mean normalized regression coefficients for TTC_v,_ *D*_v,_ TTC_A_ and 1/*L*_*A*_ were significantly positive across participants in both the IV and the NV group. Thus, participants on average based their TTC estimations on both auditory and visual information, compatible with previous studies [34,38]. Also, the estimated TTCs were associated with both the presented visual and auditory TTCs, and the distance at occlusion and the final sound level. An rmANOVA on the mean normalized regression coefficients with predictor as the within-subjects factor and vision group as the between-subjects factor showed a significant effect of predictor, *F*(7, 322) = 42.23, $\tilde{\varepsilon }$ = 0.50, *p < *.001, $\eta_{p}^{\text{2}}$ = 0.48. The predictor × vision group interaction was not significant, *F*(7, 322) = 1.72, $\tilde{\varepsilon }$ = 0.50, *p = *0.16. The mean GDW was highest for TTC_V_, followed by *D*_*v*_ and 1/ϴ (right panel in Fig 5). The GDWs were generally lower for the auditory than for the visual cues, with the highest GDW among the auditory cues observed for TTC_A_ and *D*_*A*_, while the GDW for *1/L*_*A*_ was surprisingly small. An rmANOVA on the normalized GDWs showed a significant effect of predictor, *F*(7, 322) = 471.4, $\tilde{\varepsilon }$ = 0.41, *p < *.001, $\eta_{p}^{\text{2}}$ = 0.91. For the IV group, the mean GDW for TTC_V_ was lower than for the NV group, while for 1/ϴ, the opposite relation was observed. The predictor × vision group interaction was significant, *F*(7, 322) = 3.01, $\tilde{\varepsilon }$ = 0.41, *p = *.035, $\eta_{p}^{\text{2}}$ = 0.06, albeit with a very small effect size. This is compatible with the expected higher importance of “heuristic” cues for the IV group. Pairwise comparisons between the NV and the IV group per predictor showed a significantly higher GDW for TTC_v_ in the NV compared to the IV group, and significantly lower GDWs for *v*_*V*_ and *v*_*A*_ for the NV group. All other pairwise comparisons were non-significant. The pattern of variable importance in the AV condition shown by *p*_Lasso_ (right panel in Fig 6) was consistent with the GDWs regarding the high relative importance of TTC_V_ and *D*_*V*_, as well as regarding the involvement of both auditory and visual cues. For 1/ϴ, *p*_Lasso_ showed a large difference between groups, in the same direction as indicated by the GDWs. For the auditory cues, *p*_Lasso_ was higher for TTC_A_ and lower for 1/*L*_*A*_ for the NV compared to the IV group. The GDWs showed an effect in the same direction, but with much smaller differences. Tests for effects of vision group were conducted with a hierarchical logistic regression model (same variant and parameters as for the V condition). In the AV condition, *p*_Lasso_ for TTC_V_ was 1.0 for all participants in the NV group and the zero variance in this condition resulted in convergence problems. For this reason, the predictor TTC_V_ was excluded from the analyses of effects of vision group in the AV condition. The analysis showed a significant predictor × group interaction, *F*(6, 266.3) = 2.16, *p = *.048, confirming the difference in cue importance between the two vision groups indicated by the GDWs. Pairwise comparisons between groups per predictor showed a significantly smaller value of *p*_Lasso_ for 1/ϴ in the NV compared to the IV group. All other pairwise comparisons were non-significant.

**Fig 7:**
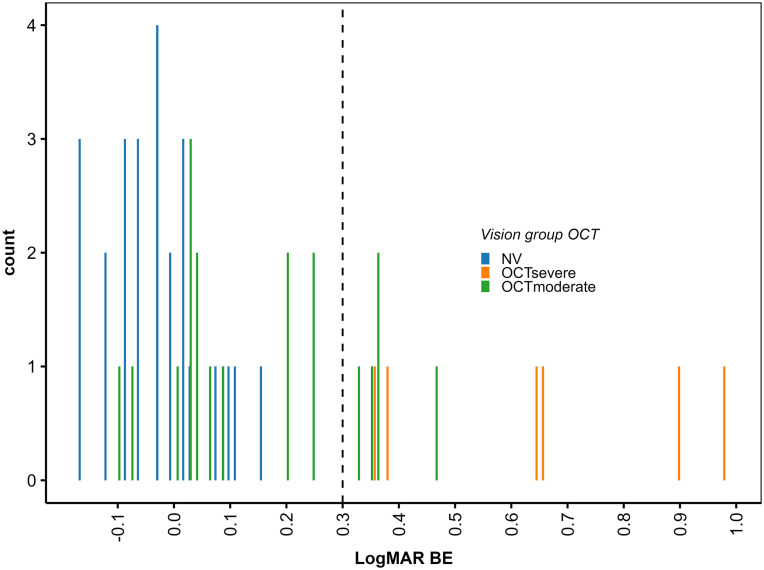
Histogram of visual acuity (log MAR) on the better eye. Blue: NV group. Orange: OCT_severe_ group. Green: OCT_moderate_ group.

Taken together, we found some significant differences in the cue weights and the relative importance of the predictors between the NV and the IV group. In most cases, the direction of these differences was as expected, but the differences were generally smaller than we had expected. One potential reason for the relatively weak group differences might be that within the IV group, the degree of vision impairment caused by their AMD was too small in some of the participants to substantially affect their visual TTC estimation abilities. To obtain at least a preliminary insight into this question, we conducted additional analyses for groups of participants defined on the basis of Optical Coherence Tomography (OCT) results, which is typically viewed as the gold standard for measuring retinal damage [85,86]. Within the IV group, we defined two subgroups of participants. Participants with retinal damage in the foveae of both eyes were assigned to the group *OCT*_*severe*_ (*N *= 6). The remaining participants in the IV groups, that is, participants for whom the retinal damage did not involve both foveae, we assigned to the group *OCT*_*moderate*_ (*N *= 17 for A and AV; *N* = 18 for V). Fig 7 shows that the visual acuity on the better eye as measured with Snellen eye charts was worse than LogMAR = 0.3 (i.e., the criterion for driving without corrective lenses in the US) for all participants in the OCT_severe_ group, while it was within the range of visual acuities exhibited by the NV group for several participants in the OCT_moderate_ group. This analysis corroborated our presumption that TTC estimation performance of the OCT_severe_ group might differ more strongly from the NV group than the TTC estimations of the OCT_moderate_ group.

**Fig 8:**
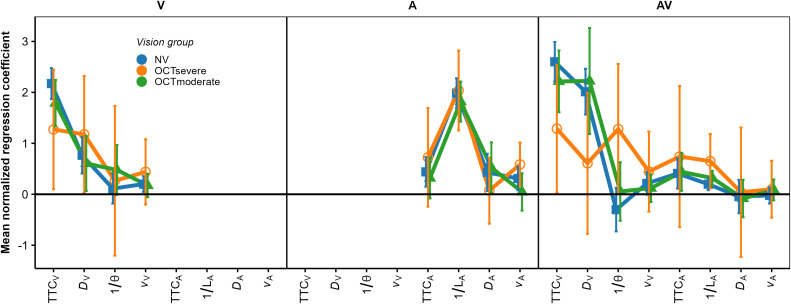
Mean normalized regression coefficients from OLS regression for V (left), A (middle), and AV (right) conditions. Blue squares: NV group. Orange circles: OCT_severe_ group. Green triangles: OCT_moderate_ group. Error bars show 95% confidence intervals (CIs).

In fact, as show in Fig 8, the patterns of mean normalized regression coefficients in the V and AV condition were more similar between the NV and the OCT_moderate_ group than for the NV group compared to the OCT_severe_ group. We compared the normalized regression coefficients for the NV (*N* = 25) and the OCT_severe_ group (*N* = 6) using an rmANOVA approach that is valid for unbalanced designs [87–89]. In the A and the V conditions, the predictor × group interaction was not significant. In the AV condition, there was a significant predictor × group interaction, *F*(7.11, 61.67) = 3.98, p = 0.001. For the NV group, the normalized regression coefficient was higher for TTC_v_ and *D*_*V*_ than for the remaining cues, while for the OCT_severe_ group, the normalized coefficients were more uniform.

**Fig 9:**
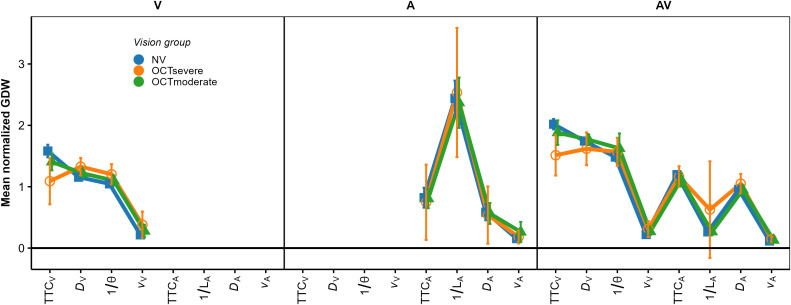
Mean normalized general dominance weights (GDWs). Same format as Fig 8.

The mean normalized GDWs were also more similar between groups NV and OCT_moderate_ than between groups NV and OCT_severe_ (Fig 9). The predictor × group interaction was significant in the V condition, *F*(1.78, 11.87) = 4.02, *p* = 0.007. In the OCT_severe_ group, the mean normalized GDW was significantly lower for TTC_V_ and significantly higher for *D*_v_ compared to the NV group. This is again compatible with the expected stronger association between the estimated TTC and “heuristic” cues in the vision-impaired group. In the A condition, the patterns of GDWs were similar across groups and the predictor × group interaction was not significant. In the AV condition, the lower observed GDW for TTC_V_ and the higher GDW for 1/*L*_*A*_ in the OCT_severe_ group compared to the NV group is compatible with the expected higher importance of auditory information in the OCT_severe_ group, but the predictor × group interaction was not significant (*p = *0.098). Note that due to the small size of the OCT_severe_ group (*N* = 6), we had low power for detecting a predictor × group interaction.

**Fig 10:**
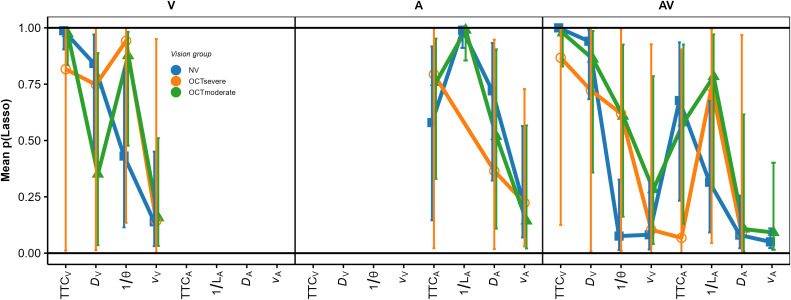
Average values of *p*_Lasso_ per predictor and vision group, plotted separately for each modality condition. Means and 95% confidence intervals were estimated using intercept-only hierarchical logistic regression models with a random intercept (see text). Same format as Fig 8.

For *p*_*Lasso*_, the estimated average values showed very large confidence intervals for the OCT_severe_ group (Fig 10), presumably due to the small group size of *N* = 6. Thus, we refrain from interpreting these results. Taken together, analyses of the cue weights and their relative importance for a subgroup of the IV group with the most severe foveal damage confirmed some of the small differences between the IV and the NV group reported above and suggests that persons with particularly severe vision impairments due to AMD might exhibit even stronger differences in their visual and audiovisual TTC estimation strategies compared to persons with normal vision.

### Analysis of mean estimated TTCs and their intraindividual variability

In addition to the analyses of cue weights, we analyzed the mean estimated TTCs and their intraindividual variability (standard deviation; *SD*) with analyses of variance. The same individual data as for the analysis of cue weights were excluded. Also, as for the analysis of cue weights, trials with technical problems (184 trials) or when the participant responded before the vehicle disappeared in the V or AV conditions (117 trials) were excluded from the analysis. Before analyzing the mean estimated TTCs, we examined the data in each modality condition for trials with implausible values of the estimated TTC, that is, where the value in the data file likely represented a response error (e.g., lapse of attention, wrong response button pressed), rather than the participant’s estimate of the TTC. To identify attentional lapses (resulting in very long estimated TTC values) and other implausible responses, estimated TTCs higher than 15 s were excluded (70 trials). Then “outliers” in the estimated TTCs were identified for each participant in each combination of modality condition, TTC level (see Table 4), velocity level (see Table 5), and source intensity (where applicable).

To define the velocity levels for this outlier detection, 20 and 30 km/h trials were pooled to create a low velocity condition, and 50 and 60 km/h trials were pooled to create a high velocity condition. All trials on which either *v*_V_ or *v*_A_ was 40 km/h were excluded from the analysis because this speed was included in both the lower and the higher group of velocities (see Table 5). For each combination of participant, modality condition, TTC level, velocity level (excluding the 40 km/h-speed), and source intensity (where applicable), a Tukey criterion [90] was used to detect “outliers” in the estimated TTCs (i.e., implausible values of the estimated TTC), excluding trials in which the estimated TTC was more than 3 times the interquartile range below the first quartile or above the third quartile. That process led to the exclusion of 98 trials (2.90% of the trials used in that outlier detection process) in the A condition (across participants: range = 0–4, *Mdn* = 2), 30 in the V condition (range = 0–4, *Mdn* = 0, percentage of excluded trials = 0.87%) and 83 trials in the AV condition (range = 0–9, *Mdn* = 1, percentage of excluded trials = 0.81%). If the exclusion of these implausible estimated TTCs resulted in no valid trials remaining for a given participant and modality condition, the participant was removed from the analysis of that modality condition. For example, one participant had to be excluded in the A condition but was included in the V condition. Accordingly, one participant was excluded when comparing the mean and *SD* of the TTC estimation in the three modality conditions, and two participants were excluded in the specific analysis of the *SD* of the estimated TTC in the AV condition. For each modality condition separately, the performance measures (mean and intraindividual *SD* of the estimated TTCs) were computed for each combination of participant, TTC level, velocity level, vehicle size (where applicable), and source intensity (where applicable).

#### Analyses of each modality condition separately.

**Fig 11:**
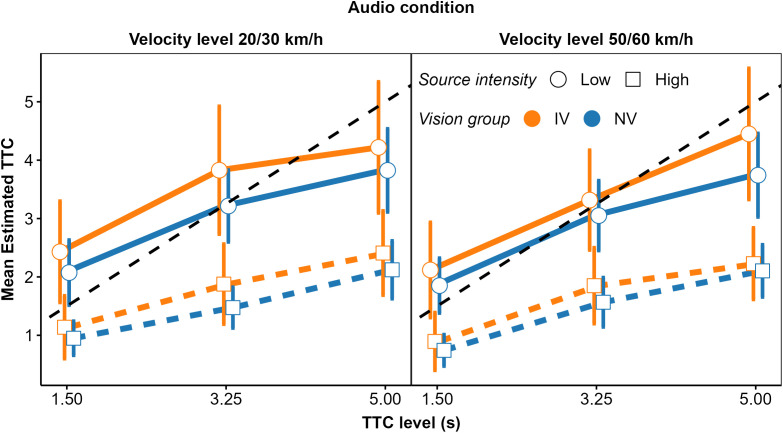
Mean estimated TTC in the auditory condition as a function of actual TTC level (in abscissa), velocity level (panel), source intensity (point shape and line type), and vision group (colors). Error bars represent the 95% confidence interval of the mean.

**Fig 12:**
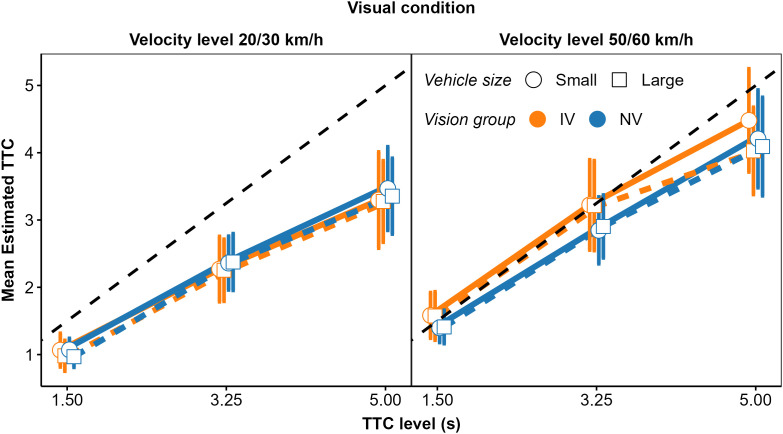
Mean estimated TTC in the visual condition as a function of actual TTC level (in abscissa), velocity level (panel), vehicle size (point shape and line type), and vision group (colors). Error bars represent the 95% confidence interval of the mean.

**Fig 13:**
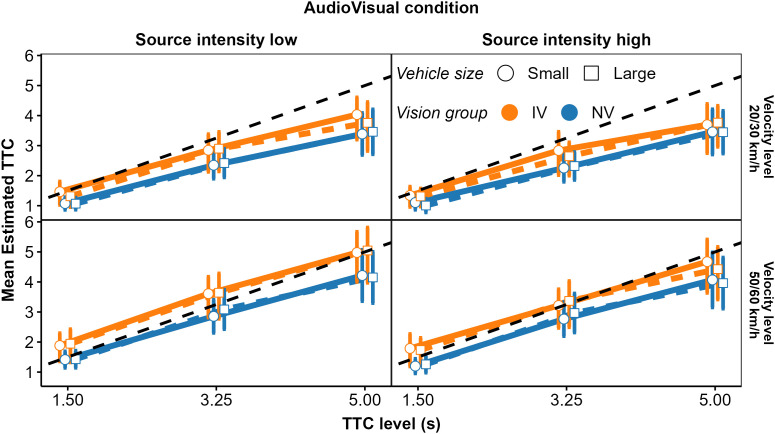
Mean estimated TTC in the audiovisual condition as a function of actual TTC level (in abscissa), velocity level (row), source intensity (column), vehicle size (point shape and line type), and vision group (colors). Error bars represent the 95% confidence interval of the mean.

The mean estimated TTCs for each modality condition are shown in Figs 11–13. Within each modality condition, a separate rmANOVA was used to analyze the mean estimated TTCs. In the V condition, the within-subject factors were the TTC level at occlusion (1.5 s, 3.25 s, 5.0 s as explained above), the velocity level (slow, fast), and the vehicle size (small-car, large-truck). The vision group (IV, NV) was a between-subjects factor. The same rmANOVA design was used for the A condition except that vehicle source intensity (soft, loud) replaced the vehicle size. For the analysis of the AV condition, all factors were included.

#### Analyses of all modality conditions together.

Another analysis compared the mean estimated TTC (and their intraindividual *SD*s) across the different modality conditions, A, V, and AV. However, because vehicle size is unique to the V condition whereas source intensity is unique to the A condition, we averaged over all vehicle size × source intensity combinations before conducting a 2 (group: IV, NV) × 3 (modality: A, V, AV) × 2 (velocity level: slow, fast) × 3 (TTC level: 1.5 s, 3.25 s, 5 s) rmANOVA.

### ANOVA results on the mean and standard deviation of the TTC estimates

Given our main interest in comparing how visual impairment influences TTC perception, we present only main effects of size, source intensity, velocity level and TTC level, and focus on the significant effects showing a main or interaction effect of the vision group.

#### Modality-specific ANOVAs.

In the A condition, mean TTC estimates increased as TTC level increased, *F*(2, 92) = 143.26, *p *< .001, $\eta_{p}^{\text{2}}$ =.76, $\tilde{\varepsilon }=.\text{6}\text{1}$ (Fig 11). Compatible with prior studies, the estimated TTCs showed a central tendency pattern (regression to the mean). The range of variation in the estimated TTC was smaller than the range of variation in the presented TTC [17,34,37,38].

Mean TTC estimates were significantly shorter for the higher velocity level, *F*(1, 46) = 4.19, *p* < .046, $\eta_{p}^{\text{2}}$ =.08 (Fig 11). This is opposite from what is typically found in the visual modality [37,91]. The few studies of TTC estimates in A only also found results different from the V modality. For example, [38] reported longest and shortest mean estimates at 30 km/h and 10 km/h, respectively, and intermediate mean estimates at 50 km/h. A previous study [37] did not find an interaction between modality and velocity.

Mean TTC estimates were significantly smaller for the higher source intensity (Fig 11), *F*(1, 46) = 127.96, *p* < .001, $\eta_{p}^{\text{2}}$ =.73, with a large effect size. This is consistent with the intensity-arrival effect found in prior studies [26,34,37,38,84,92] and the high relative importance of the sound level at occlusion in the regression analyses reported above (Fig 5). TTC level, velocity level, and source intensity had a similar effect on the intraindividual *SD* of TTC estimates; *SD* increased as TTC level increased, *F*(2, 92) = 14.41, *p* < .001, $\eta_{p}^{\text{2}}$ =.24, $\tilde{\varepsilon }=\text{1}$, was lower at the higher velocity level, *F*(1, 46) = 5.88, *p* = .019, $\eta_{p}^{\text{2}}$ =.12, and lower at the higher source intensity, *F*(1, 46) = 39.03, *p* < .001, $\eta_{p}^{\text{2}}$ =.46. This is an expected result because the intraindividual variability of estimated TTC was consistently reported to increase with the mean estimated TTC (e.g., [61,93]). The mean estimated TTC and the intraindividual *SD* of the estimated TTC did not differ significantly between the two vision groups. Thus, compatible with the regression results above, the NV and the IV group performed similarly in the A condition.

For the V condition (Fig 12), mean TTC estimates increased as TTC level or velocity level increased and was lower for the larger vehicle size (TTC level: *F*(2, 94) = 204, *p* < .001, $\eta_{p}^{\text{2}}$ =.81, $\tilde{\varepsilon }=.$61; velocity level: *F*(1, 47) = 113.24, *p* < .001, $\eta_{p}^{\text{2}}$ =.71; vehicle size: *F*(1, 47) = 11.39, *p* = .001, $\eta_{p}^{\text{2}}$ =.19). The effect of velocity level (higher mean estimated TTC at higher speed) is consistent with the high relative importance of distance at occlusion in the regression analyses reported above (Fig 5), because at a given TTC, fast vehicles were further away than slower vehicles. It is also consistent with previous results [61,91]. The effect of vehicle size is consistent with prior demonstrations of the effects of optical size [35,94]. There was a significant interaction between vision group and velocity level, *F*(1, 47) = 4.84, *p* = .032, $\eta_{p}^{\text{2}}$ =.09. The mean estimated TTC was higher at the fast velocity level compared to slow velocity level, consistent with previous results (e.g., [37]), and this effect of velocity level was stronger in the IV group compared to the NV group, consistent with the higher importance of “heuristic” cues in the IV group for the V condition shown by the regression analyses above.

Increases in TTC level and velocity level also corresponded to increases in the intraindividual *SD* of the TTC estimates (TTC level: *F*(2, 94) = 66.54, p < .001, $\eta_{p}^{\text{2}}$ =.59, $\tilde{\varepsilon }$ =.85; velocity level: *F*(1, 47) = 58.14, *p* < .001, $\eta_{p}^{\text{2}}$ =.55). Increases in vehicle size however corresponded to a decrease of the intraindividual *SD* of the TTC estimates, *F*(1, 47) = 8.29, *p* = .006, $\eta_{p}^{\text{2}}$ =.15. This is again compatible with the positive association between the intraindividual *SD* and the mean of the estimated TTC reported in the literature. Vision group had no effect on the *SD* of the TTC estimates.

In the AV condition, the mean estimated TTC (Fig 13) and the intraindividual *SD* of the TTC estimates increased with increases in TTC level, *F*(2, 92) = 172.15, *p* < .001, $\eta_{p}^{\text{2}}$ =.79 and *F*(2, 90) = 71.31, *p* < .001, $\eta_{p}^{\text{2}}$ =.61, respectively. An increase in velocity level led to higher mean estimates (*F*(1, 46) = 140.25, *p* < .001, $\eta_{p}^{\text{2}}$ =.75) and higher *SD* (*F*(1, 45) = 17.94, *p* < .001, $\eta_{p}^{\text{2}}$ =.28). A high source intensity decreased both the mean and *SD* of the estimates, respectively *F*(1, 46) = 50.99, *p* < .001, $\eta_{p}^{\text{2}}$ =.52 and *F*(1, 45) = 8.17, *p* = .006, $\eta_{p}^{\text{2}}$ =.15, again compatible with the high relative importance of distance at occlusion in the regression analyses reported above (Fig 5). Finally, the vehicle size did not significantly influence the mean TTC estimates, *F*(1, 46) = 0.008, *p* = .92, but larger size led to an increase in the *SD* of the estimates, *F*(1, 45) = 7.34, *p* = .01, $\eta_{p}^{\text{2}}$ =.13. When analyzing the mean TTC estimates, there was a significant interaction between vision group and vehicle source intensity, *F*(1, 46) = 8.15, *p* = .006, $\eta_{p}^{\text{2}}$ =.15, showing a larger difference between the groups at the lower source intensity. There also was a significant interaction between vision group, vehicle size, vehicle source intensity, TTC level, and velocity level, *F*(2, 92) = 5.66, *p* = .005, $\eta_{p}^{\text{2}}$ =.11, $\tilde{\varepsilon }$ = 1 (Fig 9).

To further understand the complex interaction involving all factors (vision group, vehicle size, vehicle source intensity, TTC level, and velocity level) influencing the mean TTC estimates, we conducted separate three-way ANOVAs at each TTC level (i.e., 1.5, 3.25 and 5 s). When TTC level was 1.5 s, there was a significant interaction between vision group and velocity level, *F*(1, 46) = 4.36, *p* = .042, $\eta_{p}^{\text{2}}$ =.09, with the IV group having a significantly higher TTC estimate than the NV group but only at the higher velocity level. There was a significant interaction among vision group, vehicle size, vehicle source intensity, and velocity level, *F*(1, 46) = 4.12, *p* = .048, $\eta_{p}^{\text{2}}$ =.08, which is too complex to interpret. When TTC level was 3.25 s, there were no main effects or interactions involving vision group. When TTC level was 5 s, there was an interaction between vision group and vehicle source intensity, F(1, 46) = 8.19, *p* = .006, $\eta_{p}^{\text{2}}$ =.15, showing that only the IV group was influenced by the vehicle source intensity. There also was an interaction between vision group, vehicle size, vehicle source intensity and velocity, *F*(1, 46) = 4.23, *p* = .045, $\eta_{p}^{\text{2}}$ =.08, again too complex to be interpreted.

Analyses of the intraindividual *SD* of the TTC estimates indicated a main effect of vision group *F*(1, 45) = 5.96, *p* = .019, $\eta_{p}^{\text{2}}$ =.11, *d *= 0.43. The *SD* was larger for the IV group compared to NV. This is the only significant effect involving vision group for the intraindividual *SD*.

#### Comparing the A, V and AV conditions.

**Fig 14:**
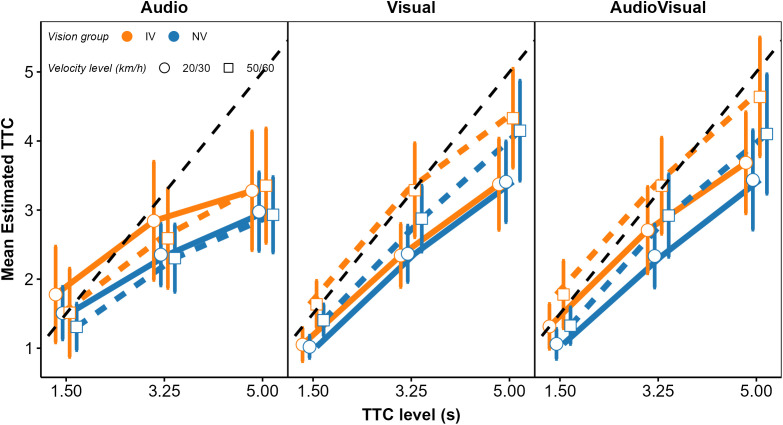
Mean estimated TTC as a function of actual TTC level (in abscissa) for each modality condition (in panel), velocity (point shape and line type) and vision group (colors). Error bars represent the 95% confidence interval of the mean.

We limit discussion to our primary interests in effects of vision group and modality. There were no significant effects or interactions involving vision group. There was a significant interaction between modality and TTC level, *F*(4, 184) = 26.85, *p* < .001, $\eta_{p}^{\text{2}}$ =.37. Compatible with previous results [38], the estimated TTCs in the A condition showed a stronger central-tendency pattern than in the conditions where visual information was available. Pairwise t-tests with Hochberg correction showed that mean estimated TTC was significantly smaller in the A condition compared to the V and AV conditions but only when TTC level was 5 s (Fig 14).

There also was a significant interaction between modality condition and velocity level, *F*(2, 92) = 67.55, *p* < .001, $\eta_{p}^{\text{2}}$ =.59. The post hoc (pairwise t tests) showed that the mean TTC estimate was significantly higher for the faster velocity level compared to slow velocity but only in the V and AV conditions (Fig 14). Longer mean estimated TTCs at higher vehicle velocity levels in the AV and V conditions but not in the A condition is consistent with previous results [38].

**Fig 15:**
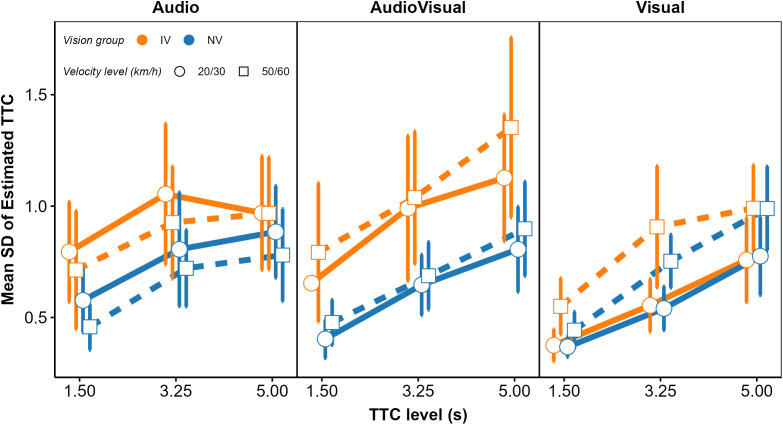
Mean *SD* of estimated TTC as a function of actual TTC level (in abscissa) for each modality condition (in panel), velocity (point shape and line type) and vision group (colors). Error bars represent the 95% confidence interval of the mean.

Analysis of the intraindividual *SD* of TTC estimates indicated a main effect of vision group, *F*(1, 46) = 4.22, *p* = .045, $\eta_{p}^{\text{2}}$ =.08, *d* = 0.27. The IV group showed more variable TTC estimations (mean *SD* = 1.03, *SD* = 0.42) than the NV group (mean *SD* = 0.81, *SD* = 0.31) (Fig 15). There also was a main effect of modality, *F*(2, 92) = 23.79, *p* < .001, $\eta_{p}^{\text{2}}$ =.34. Variability was higher in the A condition compared to the V and AV conditions. There was a significant interaction between modality and velocity level, *F*(2, 92) = 18.89, *p* < .001, $\eta_{p}^{\text{2}}$ =.29, showing that the *SD* of the TTC estimate was higher in the A modality compared to V or AV modalities, for both velocity levels (Fig 15).

**Fig 16:**
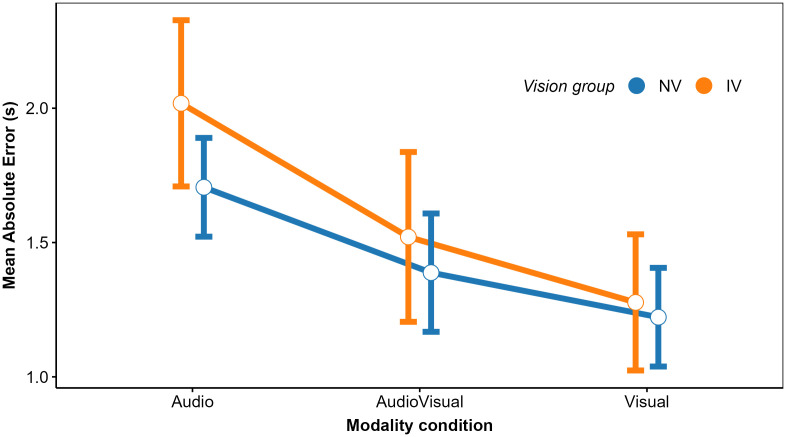
Mean absolute error as a function of modality condition (in abscissa) and vision group (colors). Error bars represent the 95% confidence interval of the mean.

We finally investigated a potential multimodal advantage of the AV condition. For that, we computed the mean absolute error (MAE; i.e., the mean of the absolute values of the difference between the estimated and the presented TTC) across all trials presented in each combination of participant and modality condition. In the AV condition, we computed the absolute error as the difference between the estimated and the presented visual TTC (Fig 6). We analyzed the MAE with a rmANOVA with modality condition as the within-subjects factor and vision group as the between-subjects factor. The results showed no significant effect of vision group, *F*(1, 46) = 1.52, *p* = .224, a significant modality condition effect, *F*(2, 92) = 25.86, *p* < .001, $\eta_{p}^{\text{2}}$ =.36, and no significant modality condition × vision group interaction. We ran separate ANOVAs to investigate potential differences between the V and AV modalities on one side, and between A and AV modalities on the other side, in both cases with vision group as between factor. When comparing the AV and V modalities, the ANOVA showed no significant effect of vision group, *F*(1, 46) = 0.38, *p* = .54, but a significant influence of modality, *F*(1, 46) = 8.57, *p* < .005, $\eta_{p}^{\text{2}}$ =.16, with a higher MAE in the AV modality (Fig 16). When comparing the AV and A modalities, the ANOVA again showed no effect of vision group, *F*(1, 46) = 2.15, *p* = .15, and a significant influence of modality, *F*(1, 46) = 20.08, *p* < .001, $\eta_{p}^{\text{2}}$ =.30, with now a higher MAE in the A modality (Fig 16). The pattern indicates that – at least in terms of the MAE – there was no multimodal advantage in the sense of a lower MAE when both auditory and visual information was available. On the contrary, the MAE was higher in the AV compared to the V condition.

**Fig 17:**
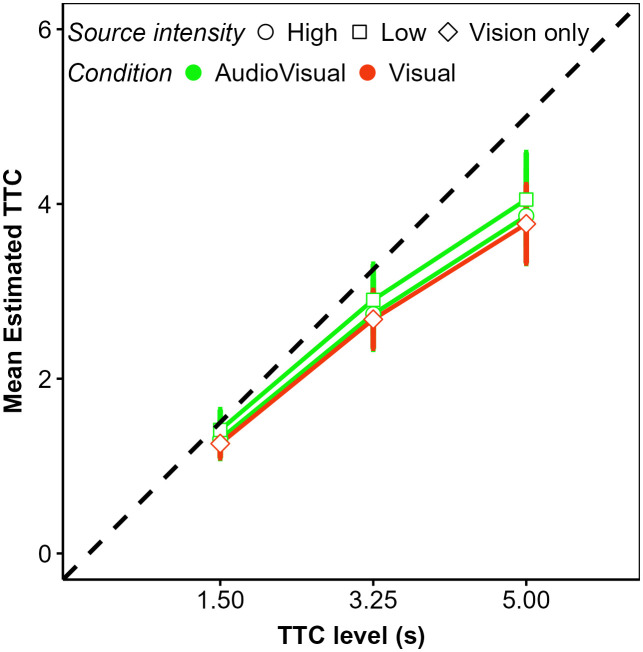
Mean estimated TTC as a function of actual TTC level (in abscissa) for the visual and audiovisual modality condition (colors), and source intensity (point shape). Error bars represent the 95% confidence interval of the mean.

Inspections of the data showed that on average, the mean estimated TTCs were closer to the veridical values in the AV compared to the V condition (Fig 17). However, the relatively strong effect of source intensity on the estimated TTCs in the AV condition increased the intraindividual variability across all trials in the AV condition. Because the MAE reflects both the deviation of the mean estimated TTCs from the veridical value (i.e., the signed error) and the intraindividual variability of the estimates (i.e., the variable error), this likely explains why, on average, the MAE was smaller in the V compared to the AV condition.

When comparing the AV and A modalities, to rule out that any difference would be due to computing the absolute error in the AV condition as the difference between the estimated and the presented *visual* TTC, we computed the absolute error in the AV condition as the difference between the estimated and the presented *auditory* TTC. The ANOVA again showed no effect of vision group, *F*(1, 46) = 2.28, *p* = .14 and a significant influence of modality, *F*(1, 46) = 13.33, *p* < .001, $\eta_{p}^{\text{2}}$ =.22, with now a higher MAE in the A modality (Fig 16).

**Fig 18:**
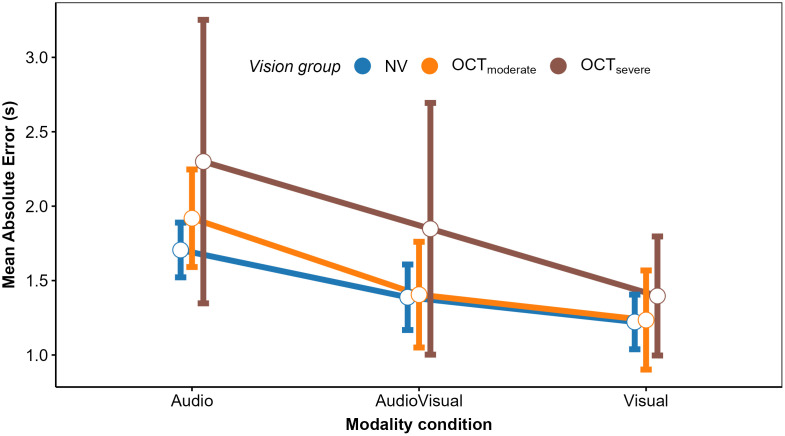
Mean absolute error as a function of modality condition (in abscissa) and vision group (colors). Error bars represent the 95% confidence interval of the mean.

Finally, to determine whether similarity in the performance of the IV and NV groups occurred because the IV group’s vision loss was not sufficient to discern differences, we compared subgroups of the IV based on the OCT results (NV and OCT_severe_), as we did for the regression analyses reported previously. Analyses of MAE were made with HRM-ANOVA tests, to cope with the highly unbalanced group sizes [87–89]. There were no significant effects of vision group (NV and OCT_severe_) or interactions with modality, likely due to high interindividual variability and small group sizes (Fig 18). Analyses of mean TTC estimates and the intraindividual *SD* of TTC estimates were conducted with HRM-ANOVAs for the A and V conditions. For mean TTC estimates in the A modality, results indicated no effect of vision group. In the V modality, results indicated a non-significant trend toward an interaction between velocity and vision group (*p* = .09) with a stronger effect of velocity in OCT_severe_ than NV, compatible with the higher GDW for *D*_*V*_, 1/ϴ, and velocity, in OCT_severe_ than NV. For the intraindividual *SD* of TTC estimates in the A modality, results suggested an interaction between vision group and TTC_A_ (*p* = .06) with a higher intraindividual *SD* of TTC estimate for OCT_severe_ than NV but only at the shortest TTC. In the V modality, results suggested a three-way interaction among vision group, TTC_V_ and vehicle size (*p* = .07), too complex to break down.

Since the HRM R-package can process only up to three within-subjects factors, HRM tests could not be used to analyze mean estimated TTC and the intraindividual *SD* of estimated TTC in the AV condition, which included four within-subjects factors. Instead, a traditional repeated-measures ANOVA (univariate approach) was used and thus results should be considered with caution due to the unbalanced group sizes. Results indicated significant interactions involving vision group but when followed up with pairwise comparisons significant differences were not obtained, likely due to limited sample size. In those conditions where there is, descriptively, a high similarity in the performances of NV and OCT_moderate_ but greater difference between NV and OCT_severe_, more OCT_severe_ participants would be required to detect a significant difference if there was one.

## Discussion and conclusions

We measured TTC estimation in individuals who had AMD in both eyes (impaired vision cohort; IV), and in individuals who did not have AMD but had normal vision (NV). We expected differences between the two groups in terms of relative weights and importance of visual and auditory cues, and in terms of performance as indicated by mean estimated TTC and intraindividual variability in estimated TTC. We also began to examine differences between the NV group and subgroups of IV that differed in severity of visual impairment because we included patients across the entire spectrum of AMD which represents a range of visual dysfunction from minimal to severe.

Our first objective was to determine to which extent the impaired vision cohort (IV) relied on their hearing when making TTC judgments in the AV modality condition where both visual and auditory cues were available. Our expectation that the IV group would rely at least partially on their residual vision rather than solely on auditory information was supported: TTC estimation was not determined by auditory information alone; visual information also contributed to such judgments. This was true for both groups as indicated by significantly non-zero regression coefficients for both visual and auditory cues (Fig 4). Conversely, the NV group also used auditory information. The two complementary measures of variable importance (GDWs from the OLS regression and *p*_Lasso_ from the cross-validated regularized regression) did not indicate a substantially higher weight on auditory compared to visual cues for the IV compared to the NV group (Figs 5–6).

Our second objective was to determine whether individuals with partial vision loss achieve better collision judgments when they have auditory information in addition to their residual vision, compared to residual vision alone, and how average performance (mean estimated TTCs and intraindividual variability of the estimated TTCs) differs from the NV group. We expected that due to their visual impairment, the IV group would exhibit less accurate and less precise TTC estimates than the NV group in the V condition. This difference was not observed. In the V condition, we found only one and relatively weak significant effect of vision group on mean estimated TTC (stronger effect of vehicle speed on the mean estimated TTC in the IV group). In the AV condition there was an effect of vehicle source intensity only in the IV group when TTC was 5 s. When TTC level was 1.5 s, the IV group showed a higher mean TTC estimate than the NV group at the higher velocity level. The only effect of vision group on intraindividual *SD* of the TTC estimates was a larger *SD* for the IV group compared to NV in the AV condition. There was no effect of vision group on the mean absolute error (MAE) in the AV condition.

When we compared the MAE in the AV condition to the V and A conditions, we did not observe a multimodal advantage in either group. In particular, the IV group did not perform better when they had auditory information in addition to visual information compared to visual information alone. The mean absolute error in the AV condition was about the average of the A and V conditions. The MAE was highest in the A condition and lowest in the V condition (Fig 16).

Our third objective was to measure how much weight the IV group assigns to reliably accurate information compared to less reliable heuristics during collision judgments, and to compare these relative weights to the NV group. Our expectation that TTC estimation in the IV group would reflect a greater importance of “heuristic cues” than reliable cues (i.e., TTC) than for the NV group was mostly supported. In the V condition, the GDWs (Fig 5) indicated a higher relative importance of distance and optical size, and lower importance of TTC_v_ in the IV group compared to the NV group. The other measure of variable importance, *p*_Lasso_, showed a different pattern, however (Fig 6). For the predictor TTC_v_, it was almost identical between groups, while for *D*_V_ and 1/ϴ, the average *p*_Lasso_ was similar between the NV and the IV group, but with a different ordering of the latter two variables. In the AV condition, TTC estimation in both groups was influenced by both visual and auditory cues and was not determined solely by auditory and visual TTC; judgments were associated with “heuristics” such as distance or the sound level at occlusion. The patterns of relative cue importance were different between vision groups, compatible with the expected higher importance of “heuristic” cues for the IV group, although the effect size was small. For example, there was a higher GDW for TTC_v_ in the NV compared to the IV group, and significantly lower GDWs for *v*_*V*_ and *v*_*A*_ for the NV group.

Consistent with our previous report of an “intensity-arrival effect” [26,34,37,38,84,92], mean TTC estimates were smaller for the higher source intensity and the regression analyses showed a high relative importance of the sound level at occlusion. Note that the 15-dB variation in source intensity can be considered large, corresponding to the loudness of the vehicles with higher and lower source intensity differing by a factor of approximately 2.5 [95]. This large variation in source intensity is within the range exhibited by real vehicles such as a large truck versus a small passenger vehicle, while probably already on the higher side. For instance, [38] reported a difference of 4 and 6 dB, respectively, in the equal loudness level of a small passenger car with combustion engine and an electric car at 30 and 50 km/h. The large variation in source intensity might have contributed to the high importance of the sound level at occlusion shown by the regression analyses. It would be interesting to study how the relative weights change when the difference in source intensity is reduced.

One potential explanation for the surprisingly similar cue weights, mean estimated TTCs, and mean absolute errors in participants with AMD compared to those without AMD is that contrary to our initial assumption, central vision, and, by implication, high visual acuity, is not universally necessary or solely responsible to extract the information needed for visual TTC judgments. Although expansion tau is an effective cue to TTC only if the rate of expansion is above detection threshold, and the looming detection threshold was reported to be higher when an approaching object is in extrafoveal compared to foveal vision [12], results from measurements of looming detection thresholds may not generalize to TTC estimation when the expansion rate is well above the threshold. In our stimuli, in most combinations of TTC at occlusion and vehicle speed, the final change rate of the visual angle ϴ (as defined in the results section) subtended by the car was well above the looming detection threshold for extrafoveal vision reported by [12]. Note also that the stimulus duration in [12] was only 200 ms, whereas we presented the approaching cars for 3 s. The considerably longer time available for integrating the expansion information will have resulted in a higher accuracy for perceiving the optical expansion. In fact, [96] measured TTC discrimination thresholds as a function of eccentricity, with a presentation duration of 1 s, and reported that the effect of eccentricity on the TTC discrimination thresholds was substantially weaker than the effect of eccentricity on visual acuity. More important, in [12], the objects were on a direct collision course with the observer, whereas in the street-crossing situation we investigated, the vehicles were on a pass-by trajectory, so that the types of visual information that were available differed from [12]. For instance, optical expansion tau is less informative for the pass-by scenario we investigated than a tau-variable computed for the azimuthal position of the vehicle (i.e., the angle between the left side of the road from the participant’s point of view and the left front corner of the vehicle). This azimuthal angle, but also other visual distance cues such as the height in the visual field might be quite robust to impairments in visual acuity. Additional research is needed to identify the effects of reduced visual acuity or using peripheral rather than central vision on such visual cues to TTC.

Our results reveal an important attribute of TTC estimations in individuals with AMD. Although their performance on the TTC estimation task in terms of the mean estimated TTCs and mean absolute error was comparable to performance in the control group with normal vision, they do not appear to use exactly the same cues. Specifically, the reverse-correlation analyses showed a higher importance of heuristic cues such as distance and sound level in the IV group than in the NV group. This is important because in the current study, the traffic scenario was relatively simple; there was only one vehicle approaching on a one-way road with no other traffic, and the parameters of the vehicle (e.g., size, speed, source intensity, TTC at occlusion) were limited in range compared to the real world where there is a much wider range and variation. It is not known whether our results would generalize to more complex and variable traffic conditions or instead whether group differences would be greater.

In light of the unexpected similarities between the IV and NV groups, even though all IV participants were clinically diagnosed with AMD in both eyes, we considered whether the IV group’s vision loss was not sufficient to discern differences from the NV group and conducted follow-up analyses. In fact, although the visual acuity of both eyes in the IV group was, on average, worse than 0.30 logMAR (20/40), average visual acuity in the better eye was 0.27 logMAR (20/37) and the visual acuity of several participants from the IV group was within the range of visual acuity exhibited by the NV group (Fig 7). This occurred in part because AMD represents a spectrum of visual dysfunction from minimal to severe and we enrolled patients across the entire spectrum of the disease.

In this context, it is important to note that comprehensive vision for patients incorporates multiple different functional aspects. Correspondingly, there are many different ways to assess visual function including: (1) high contrast, central visual acuity (typically referred to as simply visual acuity (VA), or best-corrected VA- hereafter referred as BCVA) which utilizes black numbers, shapes or letters on a white background, (2) central visual acuity in various lighting settings including low light (referred to as low luminance visual acuity) which also utilizes black numbers, shapes or letters on a white background, (3) central visual acuity (referred to as contrast sensitivity) which utilizes numbers, shapes or letters across a spectrum of contrast on a white background, (4) visual field (referred to as perimetry), (5) direct functional assessments such as reading speed or environment assessments, (6) and patient reported outcomes including many visual function questionnaires such as the NEI-VFQ.

Within this context, patients with AMD often experience a spectrum of challenges with daily visual function, and BCVA typically does not reflect the severity and extent of impairment experienced among patients with AMD. For example, reasonably good BCVA is often preserved late into the disease process when other measure of visual dysfunction may be quite advanced and dramatic. Overall therefore, BCVA does not adequately reflect disease progression or loss in function [97]. Individuals with AMD who have good VA nevertheless often encounter challenges performing tasks that depend on central vision, such as reading and facial recognition [98]. Consistent with this limited relationship between BCVA and extent of AMD pathology, there has been a discrepancy between anatomic and functional benefit when AMD progression was slowed, and no direct benefit on slowing VA progression has been noted [97,99,100], despite some evidence of preservation of visual field [101].

The association between measures of visual function and the extent of AMD pathology within the macula, for example from a lesion from geographical atrophy (GA), has consistently been reported to be moderate to poor with BCVA having the smallest association [102]. Even when loss in visual acuity was not severe, deficits in daily functions requiring vision were evident such as low-light vision, contrast sensitivity, dark adaptation, and reading, in part because the fovea may be spared until late in the disease progression [98]. Although AMD, including both nAMD and GA, interferes with mobility and tasks such as driving and walking [103], these have not been comprehensively studied as functional endpoints.

Within the IV group, we identified individuals with the most severe foveal and macular damage shown by retinal imaging using OCT. For all individuals in that OCT_severe_ group, the VA in the better eye was worse than a log MAR of 0.3. With the caveat of a small sample size, the subgroup of IV who had the most severe OCT classification exhibited greater differences in terms of cue weights from the NV group, compared to those with a more moderate OCT classification and may have driven the observed differences between the IV and NV groups. This suggests that people with particularly severe vision loss due to AMD may show stronger differences in which information they use to judge TTC compared to people with normal vision (i.e., the OCT_severe_ group relying more on heuristic cues than the NV group). It remains to be investigated if the critical factor for differences in the TTC estimation strategy caused by AMD is reduced visual acuity, as suggested by Fig 7, or if other characteristics such as reduced contrast sensitivity are more important, or if reliance on the different cues varies among patients. Mean TTC estimates and MAE also were comparable between the NV and OCT_severe_ groups. An important implication of these subgroup analyses is that simply having AMD in both eyes is not sufficient to predict TTC estimation differences between IV and NV. Rather it appears to be the degree of bilateral visual impairment of the AMD that matters. Nevertheless, it is still possible to perform the tasks even with severe visual impairment.

Although potential effects of vision group were the focus of our study, it seems important to briefly discuss the effects of modality condition and the motion parameters on the mean estimated TTCs and their intraindividual *SD*s, because studies comparing TTC estimation across modality conditions are scarce. These effects were largely consistent with the literature on TTC estimation. In all modality conditions, the intraindividual *SD*s increased as the TTC increased, as has been shown in prior studies (e.g., [61,93,104]). In the *A condition*, we replicated the intensity-arrival effect; TTC estimates were larger for softer source intensities [34,37,38,84,92]. In the *V condition*, mean estimated TTCs (and their intraindividual *SD*) increased as velocity increased (e.g., [37,91,105]). Faster vehicle velocities (and thus larger distances at occlusion) resulted in longer TTC estimates, consistent with the literature and the analysis of the cue weights. Mean estimated TTCs were shorter for the larger vehicle size, consistent with prior studies [35,94]. In the *AV condition*, higher source intensity resulted in shorter mean estimated TTC (intensity-arrival effect). Thus, compatible with the cue weight analyses and previous studies (e.g., [38]), TTC estimation was influenced by auditory information, even though full visual information about the motion of the vehicle was available. In the AV condition, vehicle size did not significantly influence the mean estimated TTC. In addition, a main effect of modality indicated that the intraindividual SD was higher in the A condition compared to the V and AV conditions.

### Consistency with prior studies comparing impaired and normal vision

The pattern of results from our IV and NV groups are consistent with that of our prior studies of younger and older adults with normal vision [34,37]. In particular, TTC estimates were based on both auditory and visual cues, both heuristic cues and reliable cues were associated with the estimated TTCs in both modalities, and the relative importance of heuristic cues (specifically inverse of optical size and SPL at time of occlusion) was greater in the auditory modality than in the visual modality. Moreover, a multimodal advantage was not observed. The intensity-arrival effect and the size-arrival effect that we reported in prior studies also occurred in our IV and NV groups of the current study. The current effects of velocity-- longer mean estimated TTCs at higher vehicle velocity levels in the AV and V conditions but not in the A condition, also align with our prior study [38]. A new finding in the current study is that the effect of velocity in the V condition was stronger in the IV group than the NV group consistent with a reliance on distance (heuristics).

Our current results of TTC estimation using the prediction-motion method also are consistent with prior studies that measured street-crossing decisions with a 5-point rating scale. In these prior studies, performance was comparable between people with normal and impaired vision (e.g., AMD, low vision, blind) when they judged whether there was enough time to cross a real street [106], whether they only saw the vehicles or both saw and heard the vehicles [27].

Relatedly, it was shown by [107] that street-crossing decisions were safe despite reliance on peripheral vision due to a simulated central scotoma and accuracy and reliability did not differ from a group without simulated central field loss. In [108], it was also demonstrated that judgments of how long it would take them to cross a street were comparable for individuals with AMD and a control group of similar age with normal vision; judgments were more affected by age, and familiarity with the road rather than vision loss. In these studies, impairment in visual acuity was more severe than in our IV group (mean logMAR 0.44 to 1.04; mean logMAR in the NV group: −0.15 to 0.03). In contrast to our current study, the prior studies did not measure the relative importance of the auditory and visual cues, or heuristic and reliable cues.

Our finding that both heuristic and reliable cues contributed to TTC estimations echoes that of a study in which blind or severely visually impaired (or blindfolded sighted) individuals rolled a ball to collide with a moving target and in which tau and less reliable cues may have influenced behavior [109]. However, the relative weights of different modalities and different cues within each modality were not measured.

Our results are not consistent with those of [17] who used the prediction-motion task to compare auditory TTC estimates in people with normal and impaired vision. The authors reported better performance in participants with impaired vision compared to that of normal vision although they acknowledged that their sample size of people with impaired vision was very small. Specifically, when six blind (no vision or light vision only) used auditory information to estimate the TTC estimate of a filmed approaching vehicle their accuracy was comparable to 60 normally sighted individuals who used visual information, and was more accurate when only auditory information was presented to both groups. Results of another study [27] also differed from [17]: Street-crossing decisions (using a rating scale) in individuals who were blind (i.e., light perception) exhibited less accurate and less safe decisions when only auditory information was presented compared to individuals with normal or impaired vision.

In conclusion, despite having AMD in both eyes, TTC estimations in the IV group were strikingly similar to those in the NV group and this occurred even though the two groups differed in age and hearing thresholds; perhaps such differences did not matter as much as their comparable cognitive abilities as measured by the T-MoCA scores. However, the relative importance of heuristic cues such as distance or sound level was higher in the IV group than in the NV group. Also, the IV group exhibited more variable TTC estimates than the NV group, although the patterns of cue weights was still similar overall. The implication is that comparable performance in terms of mean estimated TTCs or mean absolute errors can be achieved on the basis of different cues. In more demanding real-world situations with higher variability in vehicles’ parameters such as size, velocity, and sound level, this reliance on heuristics may result in greater performance differences, particularly in less effective performance in people with bilateral severe AMD as indicated by OCT. On a more general level, it remains to be shown which specific aspects of vision loss affect TTC estimation and how these potential effects can be explained.

## Limitations

There are several limitations to our study to consider and to address in future studies.

### Age

First, despite our objective to match the vision groups on age, the mean age of the IV group (78 yrs) was significantly greater than the mean of the NV group (68 yrs), due to challenges in recruiting participants in the NV group. However, the groups did not differ significantly on their T-MoCA-BLIND score (IV group: *M* = 19.3, *SD* = 1.84, NV group: *M* = 19.6, *SD* = 1.91). This is important because TTC estimates putatively rely in part on cognitive abilities (e.g., cognitive extrapolation, [110]). If the two groups were comparable on such abilities as indicated by the T-MoCA scores, age differences should not play a consequential role in TTC estimates.

More generally, recruitment was extremely challenging. Our IV participants were required to have AMD in both eyes and most such participants were much older. However, to complete the task they needed to see and hear the vehicles. Thus, their vision loss could not be too severe and their hearing had to be adequate to hear the vehicle sounds. This has implications for the applicability of our results to the larger population with the assumption that patients with less hearing would do even worse.

### Difference in hearing thresholds

Second, the two groups differed in their hearing levels with the IV group having modestly poorer hearing on average compared to the NV group. We made sure that the vehicles in the TTC task were sufficiently audible (suprathreshold) for all participants to support adequate sound awareness and localization, which was implemented successfully except for one participant who had received too low source intensities and was thus excluded from the analyses of the A and AV conditions. Still, apart from the obvious effect on the audibility of weak sounds, sensorineural hearing impairment affects a broad range of auditory processing capabilities such as frequency resolution and temporal acuity (e.g., [111]). The stronger (on average) hearing impairment in the IV compared to the NV group might thus have contributed to our unexpected finding that there was not a substantially higher weight on auditory compared to visual cues for the IV compared to the NV group.

### IV group had variable levels of foveal damage

Third, the extent to which the fovea was damaged was variable in the IV group. Our preliminary analysis suggests that differences in the relative contributions of different cues between the two groups depends on extent and severity of vision loss and greater severity will result in greater differences. In our group, a relatively small number of participants had “severe” loss and made it difficult to make strong comparisons.
